# Nose to brain delivery of dapoxetine-loaded PLGA nanoparticle for treatment of premature ejaculation distress in normal and diabetic rats with in silico targeting brain FOX and serotonin proteins

**DOI:** 10.1007/s00210-025-04876-4

**Published:** 2025-12-23

**Authors:** Fatma I. Abo El-Ela, Heba F. Salem, Mohamed M. Nafady, Rasha M. Kharshoum, Hanan O. Farouk

**Affiliations:** 1 Department of Pharmacology, Faculty of Veterinary Medicine, Beni-Suef University, Beni-Suef, 62511 Egypt; 2 Department of Pharmaceutics and Industrial Pharmacy, Faculty of Pharmacy, Beni-Suef University, Beni-Suef, Egypt; 3 Department of Pharmaceutics, Faculty of Pharmacy, Nahda University, Beni-Suef, Egypt; 4 Department of Pharmacology, Faculty of Veterinary Medicine, Beni-Suef University, Beni-Suef, 62511 Egypt

**Keywords:** Premature ejaculation (PE), Diabetic, Nanoparticles, Intranasal (IN), Dapoxetine, Molecular docking, FOX protein, Rats

## Abstract

Psychological and emotional sexual conditions in men include premature ejaculation. Premature Ejaculation (PE) has 4%–66% worldwide effects on self-esteem, relationships, and quality of life. Poor sexual confidence, anxiety, and sadness may worsen the issue. For this disease, dapoxetine HCl (DH) is used. In normal and streptozotocin (STZ)-induced diabetic rats, DH-loaded poly (lactic-co-glycolic acid) PLGA NP intranasal (IN) therapy was compared to standard lidocaine for DH release, entrapment efficiency, behavioural analysis, histopathological and immunohistochemistry expression, as neuroprotection inspection and proto-oncogene c-Fos (FOS) protein sequence expression. Also, in silico brain serotonin and FOS protein analyses were performed. Double-emulsion-solvent evaporation produced DH-loaded PLGA NP for IN quick-acting PE. Safe, controlled release, and effectiveness were investigated in PE rats using DH-PLGA NP. The brain’s FOS protein expression, mount, intromission, post-ejaculatory, and Ejaculation frequencies were estimated. Optimized spherical NPs were 174 nm in size, − 69.45 mV zeta potential, 98.04% entrapment efficiency, and 78.61% release after 8 h. The brain’s ejaculation centre’s rapid-acting DH prolongs intra-ejaculation, lowers FOS protein density, and normalizes hippocampus neurons. Histology and immunohistochemistry confirmed behavioural results of lower FOS protein expression and delayed ejaculation. Docking revealed that the DH ligand binds more strongly to brain protein active sites involved in premature ejaculation via rapid brain delivery bypassing the BBB via IN dosing, confirmed by behavioural analysis, histopathology, and immunohistochemistry. Using DH acutely decreased rapid ejaculation and changed contextual neuronal activity in ejaculatory network brain regions. Our results improve PE neurobiology and pharmacology research. Expression of FOS protein in DH nanoformula IN protects normal and diabetic rats in normal and diabetic premature ejaculated rats.

**Graphical Abstract:**

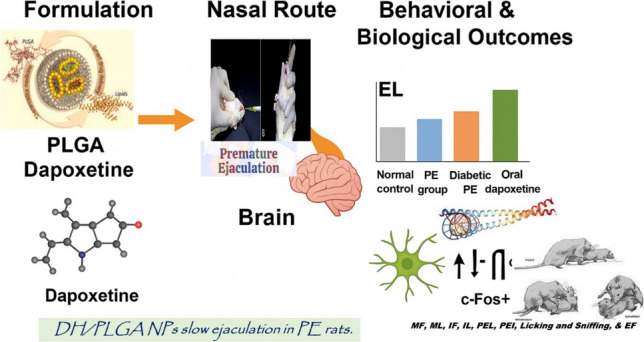

**Supplementary Information:**

The online version contains supplementary material available at 10.1007/s00210-025-04876-4.

## Introduction

PE is the most prevalent male sexual condition, affecting 4–39% of the general population. (Porst et al. 2007; Reading and Wiest 1984; Nathan 1986; Spector and Boyle 1986; Spector and Carey 1990; Grenier and Byers 1997; Laumann et al. 1999). Patients were diagnosed with PE using stopwatch studies of the intravaginal ejaculation latency time (IELT), which is the duration between penetration and ejaculation (Waldinger, et al. 2006). IELT time decreases with age and varies by nation, with a median of 5.4 min. When the IELT is less than 1 min, the patient is referred to as a PE patient (Waldinger, et al. 2006). There are two types of PE: primary (lifelong) and secondary (acquired) (Schapiro 1943). Anxiety is the main cause for the acquired (secondary) PE (Hartmann et al. 2005), psychological problems (Hartmann et al. 2005), prostatitis (Screponi et al. 2001), ED or erectile dysfunction (Laumann et al. 2005), hyperthyroidism (Carani et al. 2005), and recreational drugs (Peugh and Belenko 2001).

A disruption in glucose metabolism, insulin resistance, or insulin secretion is the cause of diabetes mellitus (DM), a metabolic disorder (Bacon et al. 2002). According to the WHO, some 220 million people worldwide are diagnosed with diabetes, and this figure is expected to quadruple by 2030 (Bacon et al. 2002; Blanco et al. 1990). The ED (erectile dysfunction), which is one of the primary reasons for the PE patient, is one of the main disorders or consequences caused by DM (Porst et al. 2007). For the past 20–30 years, behavioural psychotherapy has been the primary treatment for PE, with medication following as a secondary option (Semans 1956; Belliveau and Richter 1970; Jannini et al. 2002). Selective serotonin re-uptake inhibitors (SSRIs) are used to treat PE by blocking serotonin and 5-hydroxytryptamine (5-HT) receptors during the ejaculation process (Olivier et al. 1998; Waldinger et al. 1998; Pattij et al. 2005a). SSRIs have been shown to be safe and effective in delaying the ejaculation process in a number of earlier trials, validating their use as the first line of therapy for acquired and lifelong PE (Waldinger et al. 2004).

About dapoxetine use for PE treatment through intranasal (IN) and as nanoformulation. The first drug used to treat PE was dapoxetine HCl (DH), which is a powerful SSRI (Sorbera et al. 2004). Dapoxetine’s primary mechanism of action involves the inhibition of 5-HT reuptake, norepinephrine, and dopamine (Gengo et al. 2005). The ejaculation process is governed by both the peripheral and central neural systems, and more recently, the supraspinal nuclei and medial amygdala have been implicated in the ejaculation process, as revealed by the gene c-FOS protein expression pattern in rats (Gengo 2005). The oral administration of DH in vivo is overtly hampered by relatively hepatic first-pass metabolism, which results in its poor bioavailability (42%), despite its remarkable pharmacological characteristics (Fouad et al. 2018; Salem et al. 2020a). Customizing formulations for clinical use necessitates addressing the aforementioned obstacles. Consequently, it is imperative to enhance the efficacy of DH by implementing innovative strategies.

This investigation is designed to prepare the DH nonformula for administration via IN dosage. A critical goal is to ensure that patients are treated with fewer adverse effects of standard medications and with greater compliance. The IN administration of molecules has become an intriguing method for therapeutic delivery to the brain in the past ten years. This method is capable of traversing the blood–brain barrier of numerous exogenous molecules and enhancing patient compliance. The nasal route is alluring because it is non-invasive. There is an increasing interest in direct nasal-to-brain drug delivery due to the feasibility of preventing first-pass intestine and hepatic processing and reducing the quantity of medicinal drugs. IN administration is a safe, cost-effective, and patient-friendly alternative to the traditional method of delivering medications to the central nervous system (CNS) by circumventing the blood–brain barrier (BBB). This objective is accomplished by administering the compounds to the fissure of the bulb region in the nasal cavity using the appropriate apparatus (Crowe et al. 2018). The mucociliary clearance and the limited quantity of active molecule that can reach the brain are the primary drawbacks of IN administration. The drug concentration that reaches the brain is occasionally below the 6 therapeutic level as a result of the limited volume of the nasal canal, which leads to insufficient therapeutic brain levels and poor absorption (Kapoor et al. 2016). Many strategies have been proposed to overcome these constraints, including Illum et al. (2012)’s description of a variety of modulator absorption systems and absorption promoters that could potentially increase the dosage delivered to the brain (Illum 2012).

The polymer carrier for the DH through IN dosage is poly (lactic-co-glycolic acid) (PLGA) in this study. Biodegradable carriers have been utilized to transport medications across the mucosal barrier and/or to safeguard them from degradation in the nasal cavity, as indicated by numerous studies. Nanoparticles (NPs) are appealing drug delivery solutions for the brain because their composition, structure, size, hydrophobicity, coating, chemistry, surface charge, and ligands can be altered (Tosi et al. 2016). The utilization of polymeric NPs is perceived as a promising and enticing technique in light of the advantages and disadvantages of various drug delivery methods (Md et al. 2013; Sharma et al. 2014). PLGA, a biocompatible and biodegradable polymer, is extensively employed for the therapeutic delivery of hydrophilic and hydrophobic drugs. Polymeric NPs are produced from this polymer (Md et al. 2013). PLGA has the potential to safeguard medications from degradation in the nasal cavity. PLGA has been employed for controlled drug release purposes for decades, making it an evident choice for the encapsulation of numerous pharmaceuticals used to treat brain disorders (Sharma et al. 2014).

To the best of our knowledge, no published research has evaluated the therapeutic effects of intranasal administration of PLGA nanoparticles containing DH in an experimental model of premature ejaculation. However, previous studies have attempted to increase the efficacy and treatment of PE by combining multiple drugs, as shown by Olivier (Olivier et al. 2023). In another investigation, all of the pharmaceuticals used for PE treatment were illustrated; however, none of them were tested for the IN nanoformulation and treatment (McMahon 2016). An additional molecule, Modafinil, was initially identified as a wake-promoting agent in a separate study and is currently employed to treat narcolepsy. The mechanism of action of modafinil is intricate and inadequately comprehended, and it appears to involve the induction of alterations in brain activation (Ballon and Feifel 2006). Our research is focused on the systematic development of PLGA nanoparticles that are specifically designed to target the CNS and are intended for intravenous administration to treat premature ejaculation. The double emulsion-solvent evaporation method was employed to incorporate DH in PLGA nanoparticles using the Box-Behnken design. The DH-PLGA NPs were assessed for ex vivo drug permeation, in vitro drug release, particle size, and EE%. One of our objectives in this investigation is to resolve the issue of premature ejaculation in males by directly targeting the dapoxetine substance to the brain through passive targeting. The substance is able to reach the brain rapidly and directly through passive diffusion from the nasal tract, circumventing the blood–brain barrier, with IN administrations. The efficacy and speed of this treatment surpass those of conventional dapoxetine treatments. The rapid-acting DH and the targeted IN formula for the ejaculation centre in the brain were the primary findings of the study. These findings were confirmed by sexual behavioural tests (extension of the intra-ejaculation duration), immunohistochemistry (FOS protein density %), and histology (Hippocampus). This research is of the utmost importance in the field of sexual medicine for patients with controlled DH release who have normal or diabetic PE.

## Materials and methods

### Materials

In the form of a purified powder salt, dapoxetine HCl was generously provided by Spimaco Addwaeih (Riyadh, Saudi Arabia) without any excipients, additives, or preservatives. Obtained from the Arab pharma company Al Amriya in Cairo, Egypt, Lidocaine Topical Aerosol; puritans pride premium, Yohimbine (Yohimbe 2000^®^). The Sigma-Aldrich Co. (St. Louis, MO, USA) was contacted to order sorbitan monooleate (span 80), polyvinyl alcohol (PVA), and poly (lactic-co-glycolic acid) (PLGA) copolymer (75/25; Resomer RG 755, MW 63 600). Dialysis tubes with a molecular weight cut-off of 12,000 Da were procured from SERVA Electrophoresis GmbH (Heidelberg, Germany). Disodium hydrogen orthophosphate, potassium dihydrogen orthophosphate, potassium chloride, absolute ethanol, methanol, calcium chloride, and dichloromethane were acquired from El-Nasr Pharmaceutical, Cairo, Egypt. Every other reagent was of the highest quality to be found.

### Methods

#### Design and optimization of experiments

The Box–Behnken design was developed by the Design Expert® software (Version 11, Stat-Ease Inc. Minneapolis, MN). The design contains three levels and three factors. In order to generate PLGA NPs models, a group of 15 experimental trials was necessary. The concentration of aqueous external phase (PVA) (B), the volume of aqueous internal phase (C), and the quantity of PLGA polymer (A) were the independent variables that were examined. The dependent variables were the cumulative quantity of DH permeated/unit area in 24 h (Q24) (Y4), particle size (Y2), release over 8 h (Y3), and EE% (Y1). The independent variables that are the subject of this investigation are listed in Table 1, along with their low, medium, and high levels. These levels were selected based on the results of preliminary experiments.

**Table 1:** Variables and their corresponding levels in the employed Box–Behnken design for DH-loaded PLGA NP_S_

Variable	Design level		
	Low (− 1)	Medium	High (+ 1)
**Independent variables**			
**A = PLGA amount (mg)**	**25**	**50**	**75**
**B = PVA concentration (% w/v)**	**1**	**1.5**	**2**
**C = aqueous internal volume (ml)**	**0.5**	**0.75**	**1**
**Dependent variables**	**Constraints**		
**Y**_**1**_** = EE%**	**Maximize**		
**Y**_**2**_** = Particle size (nm)**	**Minimize**		
**Y**_**3**_** = Q**_**8h**_** (%)**	**Maximize**		
**Y**_**4**_** = Q**_**24**_** (µg/cm**^**2**^**)**	**Maximize**		

*EE% *entrapment efficiency percentage, *PLGA* poly lactide co-glycolide, *PVA* polyvinyl alcohol. Q8h, cumulative discharge after 8 h; Q24, cumulative quantity disseminated per unit area in 24 h

#### Development of DH–PLGA NPs

The double emulsion (w/o/w) method, as described by Zambaux et al. was employed to produce PLGA NPs. (Zambaux et al. 1998). The following ingredients were used to produce a variety of PLGA NPs for DH: PLGA (25, 50, and 75 mg), PVA (1, 1.5, and 2% w/v), and aqueous internal phase volume (0.5, 0.75, and 1 ml) (Abdelkader et al. 2018). Separately, PLGA was dissolved in 0.5 ml of dichloromethane (organic phase), and the internal aqueous phase was formed by adding 1% w/v Span 80 and 30 mg DH to distilled water and agitating under magnetic agitation at ambient temperature. The dichloromethane was extracted under reduced pressure after 15 min of magnetic agitation. The primary emulsion (w/o) was generated by adding organic solvent containing PLGA to the internal aqueous phase in a drop-by-drop manner using a magnetic stirrer. Subsequently, the aqueous external phase was generated by dissolving 10 ml of distilled water with PVA using a magnetic stirrer. Lastly, the primary emulsion was homogenized at 13,500 rpm for 10 min while being drizzled onto the aqueous external phase at a constant rate until the formation of w/o/w.

### Characterization of the experimental runs

## Determination of DH EE%

The EE% of DH-loaded PLGA NPs was indirectly estimated by subtracting the free DH (non-entrapped drug) from the total amount of DH that was actually added to the formulation. At 4 °C, the NPs were subjected to a cooling centrifugation (SIGMA 3–30 K, Steinheim, Germany) at 14,000 rpm for 1.5 h (Ahmed et al. 2022; Mahmoud et al. 2022). The concentration of residual free DH in the filtrate was determined spectrophotometrically at *λ*max 292 nm after an appropriate dilution. The equation below was employed to estimate the EE% of the DH NPs (1).

1 $$EE\%=\frac{Total\;drug\;amount-free\;drug\;amount}{Total\;drug\;amount}\times100$$

## Determination of particle size and zeta potential

The average NP size (z-ave), polydispersity index (PDI), and zeta potential of DH-PLGA-NP were evaluated using dynamic light scattering (DLS) in a Nano ZS Zetasizer (Malvern Instruments, Malvern, UK). Before the measurement, each sample was attenuated by the addition of deionized water. The analysis was conducted at ambient temperature (25 ± 2 °C). The mean values ± SD were determined by scanning each specimen three times.(Nagarajan et al. 2015; Salem et al. 2021).

## In vitro release study of DH-loaded PLGA NPs

An in vitro discharge study was conducted in triplicate at 32 ℃ across a cellulose dialysis membrane. In summary, various formulations of PLGA nanoparticles (equivalent to 3 mg of DH) (Salem et al. 2020b) and glass cylinders (6 cm in length and 2.5 cm in internal diameter) were introduced and sealed at one end with a dialysis membrane with a molecular weight cutoff of 12,000 Da. Overnight, the membrane was submerged in the receptor milieu. The laden cylinders were secured using the shafts of the USP dissolution tester apparatus (Abdelrahman et al. 2017; Salem et al. 2020c). Thirty milliliters of simulated nasal electrolyte solution (SNES) with a pH of 5.5 were employed as the release medium to ensure sink conditions (Aboelwafa et al. 2010). The SNES had 7.45 mg/ml NaCl, 1.29 mg/ml KCl, and 0.32 mg/ml CaCl_2_. 2H_2_O and a pH of 5.5 were also added (Cheng et al. 2002; Callens et al. 2003). The rotation speed was modified to 100 rpm, and the temperature was set to 32 ± 0.5 °C during the release research. To ensure a consistent volume, 3 ml aliquots were removed from the release medium and replaced with an equivalent volume of fresh medium at 0.5, 1, 2, 3, 4, 6, and 8 h later. The concentration of DH was determined by spectrophotometry at *λ*max 292 nm following the filtration of samples using a 0.45 m Millipore filter. The results of the release research were presented as means ± standard deviations for each formulation, and they were conducted in triplicate. The cumulative percentage of DH-loaded PLGA NPs that were emitted was plotted against time.

The mechanism of DH release from its PLGA NPs was determined by fitting the acquired data to zero-order, first-order kinetics, and the Higuchi diffusion model. The orders of release were subsequently established by determining the magnitude of the coefficients of determination (*R*^2^) in each instance and subsequently selecting the appropriate mathematical model.

## Ex vivo permeability study

The local Animal Ethics Committee of Nahda University approved this investigation. Camel buccal mucosa was obtained from a nearby slaughterhouse and utilized within one hour of the animal’s death. Distilled water was used to collect the nasal mucosa of the camel that had been recently removed, with the exception of the septum (El-Nabarawi et al. 2016a). For 30–60 min, the membrane was equilibrated in distilled water (El-Nabarawi et al. 2016a). The nasal membrane was recognized, and the superior nasal concha was subsequently isolated. The vertical Franz diffusion cell was then used to place the superior nasal membrane that had been excised. A Franz diffusion cell with a surface area of 5 cm^2^ was employed for ex vivo diffusion investigations. The nasal membrane surface was only briefly flooded with the diffusion fluid by maintaining the temperature of the chamber, which contained 100 ml of distilled water, at 37 ± 0.5 °C and agitating continuously with a magnetic bar at 100 rpm. The investigation was conducted in non-occlusive conditions. Various volumes of PLGA NP dispersion containing constant quantities of DH (3 mg) were introduced into the donor compartment of the Franz diffusion cell. At predetermined intervals (1, 2, 3, 4, 5, 6, 8, 12, and 24 h), no more than 3 ml of receptor compartment samples were extracted and replaced with equivalent volumes of new milieu. Lastly, the samples were analysed at 292 nm using a spectrophotometer after being filtered through a 0.45-m Millipore filter.

The total quantity of DH penetrated per unit area (g/cm^2^) was plotted against time (h) for each formulation. For each DH-PLGA-NP and the control DH solution, the permeation parameters Q24 in g/cm^2^, latency time in minutes, permeability coefficient (Kp) in cm/h, and drug flux (Jss) in g/cm^2^ h were determined. Additionally, the following formula was employed to calculate the enhancement index (EI) (El-Nabarawi et al. 2016b):

2 $$\mathrm{EI}= \frac{\text{Kp of PLGA NPs}}{\text{Kp of control solution}}$$

## Optimized DH–PLGA–NP characterization

### Particle morphology

The optimized DH-PLGA-NP was subjected to a morphological investigation using a JEM-1400 transmission electron microscope (Jeol, Tokyo, Japan). A drop of the Nano dispersion was deposited on a copper grid after it had been adequately diluted, and the excess was removed using filter paper. Afterward, a negative stain was applied, which was a 2% w/v phosphotungstic acid aqueous solution. The excess was also extracted. The air-dried sample was subsequently investigated using a transmission electron microscope at 80 kV at room temperature (Abdullah et al. 2016).

### Stability study of PLGA NPs

The sealed containers at 4 °C were used to retain the optimal DH-loaded PLGA NPs formulation for a period of 3 months. Samples of the optimized DH-prepared nanoformula mounted on PLGA NPs were extracted immediately following assembly and at predetermined intervals for a period of 3 months. The zeta potential, entrapment, PDI, and particle diameter of the samples were examined (Nasr et al. 2008).

### Differential scanning calorimetry (DSC)

The thermal characteristics of unadulterated DH, PLGA, PVA, and span 80, as well as (1:1) mixtures of each of these substances with DH, were assessed using DSC (DSC 50 Shimadzu, Kyoto, Japan). Initially, a conventional aluminum pan was used to deposit 5 mg aliquots, which were heated from 25 to 300 °C at a scanning rate of 5 °C/min, while 25 ml/min of inert nitrogen circulated through the pan (Menshawe et al. 2019).

### In vivo study of optimized DH-PLGA NP in yohimbine-induced premature ejaculation in normal and diabetic rats

## Animals

Adult male albino rats weighing 250–30 gm and aged 3–4 months were used in the studies (Beni-Suef University Faculty of Veterinary Medicine, Lab animal). The usual lab environment has ambient temperature, humidity, and a 12-h light/dark cycle. Daily rodent food and clean water were available 24/7 from easy sources. The Nahda University Faculty of Pharmacy’s IACUC accepted this rat research with permission number NUB-011–019. All animal care, dosing, treatment, PE induction, weighing, and injections followed the protocol.

## Treatment design

Seven equal groups of six mature male Albino rats (250–300 g, 3–4 months) were formed from forty-two animals. Rats in the control negative group (CNT) (G1) received distilled water. Group two (G2) (control positive, PE, non-treated) rats received an oral Yohimbine solution at 18 mg/200 gm b.wt., corresponding to 1000 mg/kg/human, according to the capsule manufacturer’s recommendations (Clark and Stanford University Department of Physiology 1983). Each rat in G3 received Streptozotocin 50 mg/kg b.wt., I/p, for diabetic induction according to Ahmed et al. (2019). After Yohimbine-induced PE, G4 rats got standard spray on the glans penis and IN lidocaine. G5 rats received 20 µL (30 mg)/kg dapoxetine nano-preparation IN after Yohimbine-induced PE and before entering the sexually prepared female rats. G6 rats were given the same dapoxetine IN formula as diabetic rats. Rats were given dapoxetine NPs and yohimbine in both nostrils while supine without anaesthesia. Finally, G7 rats got usual oral dapoxetine powdered not prepared. One stomach tube gavage was used daily for all treatments, dosages, and routes, including control. Experimental design for the different groups’ clearly stated in Table 2.

**Table 2:** Group’s terminology and description

X-axis label	Full term/description
Control negative	**Normal, untreated animals**
Control positive	**Premature ejaculation (PE) model, untreated (vehicle)**
Positive diabetic	**Diabetic PE model, untreated (vehicle)**
Standard	**PE model treated with a standard topical lidocaine**
Normal, PE, ttt IN	**PE model treated with the developed nanogel via intranasal (IN) administration non-diabetic rats**
Diabetic ttt IN	**Diabetic PE model treated with the developed nanogel via intranasal (IN) administration**
ttt orally	**Diabetic PE model treated with the drug via oral administration (oral)**

ttt means: treatment

After 8 weeks on a high-fat diet (HFD), rats received an intraperitoneal injection of STZ (2% STZ at 30 mg/kg) Sigma-Aldrich, USA, dissolved in citrate buffer with a pH of 4.5 and monitored at 4 °C. To cause diabetes, measurements of random blood glucose were taken 72 h following STZ treatment. RBG values below 16.7 mmol/L were thought to indicate diabetes in rodents. A random assessment of HFD/STZ-induced diabetic rats was performed. Height and weight fasting blood glucose (FBG) levels were measured weekly. Rats take HFD until 12 weeks, when they get ketamine and xylazine under anaesthesia. Plasma from peripheral blood from the inner canthus was centrifuged at 3000 RPM for 10 min, and glucose was tested. A serum was then obtained.

## Experimental design

Sexually receptive females were prepared as method previously described by Heijkoop et al. (2018) as females induced to be in oestrus through hormonal injection of oestradiol benzoate (100 μg/ml) and progesterone (5 mg/ml) powder through a mixture of the two hormones together. For usage, heat the hormonal combination at 60 °C for 1 h and shake well. Progesterone is injected 4 h before sexual activity and oestrogen 52 h before copulation. We injected both hormones at 0.2 ml each animal.

## Behaviour observations

Behaviour was tested 15 min after IN injection. Male sexually trained rats were well-maintained for 30 min following female cage entrance under a dimmed red light. The sexual activity occurred between 7 and 10 a.m. Male rats were caged under glass for sexual activity observation and video capture. Male rats were given 15 min to adjust before females arrived. Six cages received three to four oestrus-induced females since each male rat had its own cage. Digital camera recordings showed male rodent reproductive behaviour. Mount latency (ML) was measured as the time it took a male to start sex without introduction. Intromission latency is the time between male copulation’s first and second intromissions. Introduction frequency (IF) is the number of intromissions before ejaculation begins. EL is the distance between intromission and ejaculation, whereas PEI is the time between ejaculation and intromission. Each male in each group was monitored for 40 min and measured daily. The experiment ended if intromission did not occur within 10 min after coupling with females. After female ingress, intromission happened 2, 3, 4, or 5 min later or less than 10 min for precise ejaculation time calculation and confirmation. Vaginal sperm was detected after ejaculation to confirm behaviour.

## Brain tissue collection for immunohistochemistry evaluation

After the initial ejaculation and observation time, male rats were slain by spinal dislocation under anaesthesia with a 1:1 (0.1 ml/100 gm) combination of ketamine (90 mg/kg b.wt.) and xylazine (5 mg/kg). Brain tissues were quickly frozen and kept at –80 °C for analysis.

## Evaluation of the male rat sexual behaviour

Various sexualities the parameters were estimated from mounts, intromissions, and ejaculations frequencies were recorded using a scoring device during sexual behaviour assessment, such as videos. The behaviour of a sexually trained male rat makes mounting, intromissions, and ejaculations easy to see. A backward jump and a strong shove mark the introduction (Heijkoop et al. 2018). Sexual behaviour was assessed using mount latency, intromission latency, and the time between being introduced to a female and mounting for the first time. Statistics on mounting, intromissions, ejaculations, and more are presented (Heijkoop et al. 2018).

## FOS protein estimation

In the freezing microtome, brains were cut into 40-mm frontal slices. These sections were immunocytochemically analysed for c-FOS protein, as previously described (Jong et al. 2005; Yokosuka et al. 2001) in the different treated groups of rats.

## Induction of diabetes

Streptozotocin (50 mg/kg b.wt., i.p.) was dissolved in buffer citrate (pH 4.5) before delivery. For 15 days, rats were fed a fat-rich diet and glucose-rich drink to induce diabetes (Shalaby et al. 2018). The study used rats with intermediate diabetes and hyperglycemia (blood glucose 360 mg/dL) after 1 week (Shalaby et al. 2018). For the estimate, blood was taken from the tail vein.

## Tolerability and acute toxicity studies

Twenty male Wistar albino rats weighing 200–250 g were given 20 µLof each medication, including dapoxetine non-formula for PE in normal and diabetic patients, saline as a vehicle, and IN lidocaine spray for 24 h to assess nasal toxicity and safety. The nasal septum and epithelial cell membrane were removed, and the rats were slain ethically and humanely using the anaesthetic approach outlined in this paper (Tengamnuay et al. 2000; Zaki et al. 2007). After 24 h in 10% formaldehyde, the specimen was decalcified and dehydrated with ethyl alcohol. The specimens were cleaned in xylene before embedding in paraffin for 24 h at 56 °C in a hot air furnace. Slide microtone was used to make 5 mm paraffin beeswax tissue slabs for sectioning. The tissue segments were deparaffinized, mounted on glass transparencies, and stained with hematoxylin and eosin. Light microscope slides of untreated and treated tissues were studied (Bancroft and Gamble 2008).

## Histopathological examination

Brain and nasal samples were buffered formalin-fixed for 48 h. Histological procedures included washing, dehydration, paraffin embedding, microtome slicing at 5 mm thickness, and hematoxylin and eosin staining (Bancroft and Gamble 2008). Tissues were embedded in paraffin wax after going through several manufacturing processes. The embedded slabs were sectioned using a microtome with a 5–6 mm cutter and stained with haematoxylin and eosin, as is customary (H&E).

## Statistical analysis

The sample size was obtained using G*Power (version 3.1) to guarantee statistical power for group differences. A power (1–β) of 0.8 and a significance level (α) of 0.05 were used to determine the minimum animal count per group based on earlier research. Thus, 42 adult male albino rats (average body weight 250–300 g; average age 3–4 months) were split into 7 equal groups of 6 rats to meet the expected sample size. One-way analysis of variance (ANOVA) and Tukey’s post hoc test for multiple comparisons were used in SPSS (version 20.0) software (IBM SPSS Statistic 20.0, Armonk, NY, USA). Results were calculated using mean minus SEM. Statistically significant *p* values were 0.05 or less (Snedecor and Cochran 1982).

### Molecular docking

The expected proteins of brain receptors, along with their 3D and 2D binding affinities and interactions, were assessed against dapoxetine in conventional and nanoformulation forms, lecithin, and chitosan through molecular docking studies utilizing the computational software Discovery Studio (DS), Structure-Based Design program (BIOVIA Inc., San Diego, USA). The 3D SDF X-ray crystal structure of the ligands was obtained from the PubChem website. A homology model of the tested receptors was furnished by the UniProt site and the protein data bank. The procedure was performed as discussed previously (Abou-Taleb et al. 2024), and the details of the study methodology were depicted in the supplemental file.

## Results

### Characterization of PLGA NPS

#### DH EE%

One remarkable feature of PLGA NPs that supports its prospective use as an intranasal delivery method is its ability to entrap high amounts of DH. As reported in Table 3**,** the EE% fluctuated between 51.93 ± 0.70% and 95.29 ± 2.67%.

**Table 3:** Experimental runs, independent variables and their responses using box benkhen design for DH-loaded PLGA NPs

Formulation	Independent variables			Dependent variables			
	A: PLGA polymer amount (mg)	B: PVA concentration (% w/v)	C: aqueous internal phase volume (ml)	Y1: EE%	Y2: particle size (nm)	Y3: Q8h (%)	Y4: Q24h (µg/cm^2^)
P 1	50	2	0.5	95.29 ± 2.67	185.51 ± 15.14	55.97 ± 8.19	515.61 ± 29.41
P 2	75	1.5	1	84.37 ± 5.03	393.50 ± 21.00	79.97 ± 14.46	211.73 ± 9.72
P 3	75	1	0.75	94.49 ± 6.84	420.04 ± 19.41	24.76 ± 5.22	209.56 ± 11.69
P 4	50	1	0.5	74.42 ± 2.89	311.61 ± 22.95	46.85 ± 7.13	398.84 ± 12.91
P 5	50	1.5	0.75	61.37 ± 3.89	346.74 ± 11.32	43.53 ± 5.23	273.15 ± 13.47
P 6	50	1	1	69.55 ± 4.27	505.43 ± 25.28	50.16 ± 7.76	170.57 ± 10.36
P 7	25	1	0.75	66.72 ± 7.02	345.67 ± 10.18	37.31 ± 4.41	313.71 ± 16.84
P 8	25	1.5	0.5	89.04 ± 9.00	204.36 ± 13.07	77.37 ± 12.35	495.97 ± 20.12
P 9	25	1.5	1	51.93 ± 0.70	299.42 ± 9.01	80.70 ± 16.32	403.41 ± 17.56
P 10	50	1.5	0.75	62.79 ± 1.23	348.47 ± 17.1	46.22 ± 7.68	256.66 ± 15.58
P 11	75	1.5	0.5	92.50 ± 6.89	456.35 ± 28.34	66.22 ± 9.55	192.13 ± 8.77
P 12	75	2	0.75	93.47 ± 4.02	329.23 ± 8.35	57.15 ± 6.45	372.83 ± 14.33
P 13	25	2	0.75	80.85 ± 0.53	241.51 ± 6.35	70.14 ± 9.78	471.12 ± 23.76
P 14	50	2	1	72.38 ± 8.05	353.00 ± 14.00	74.51 ± 13.33	214.63 ± 18.28
P 15	50	1.5	0.75	64.66 ± 3.18	347.14 ± 20.10	48.61 ± 6.87	271.91 ± 16.29

*PVA* polyvinyl alcohol, *PLGA* polylactide co-glycolide, *EE%* encapsulation efficiency percentage. Q8h provides the cumulative release after 8 h (in percentages), while Q24 represents the cumulative quantity disseminated per unit area over 24 h. Mean values (*n* = 3) ± standard deviation are represented in the data

A polynomial quadratic model was found to be appropriate through statistical ANOVA analysis. The entrapment was substantially influenced by the quantity of PLGA (A), concentration of PVA (B), and volume of aqueous internal phase (C) (*p* < *0.05*). A reasonable difference between the predicted *R*^2^ (0.6813) and the adjusted *R*^2^ (0.9395) was achieved, resulting in an adequate precision of 15.05. Consequently, the three independent parameters significantly influenced the EE% values of DH-loaded PLGA NPs (*p* < *0.05*). Equation illustrates the regression-coded equation for EE% (3).

3 $$\mathrm{EE\%}=\text{62.94 + 9.54 A + 4.60 B }-\mathrm{ 9.13 C }-\text{ 3.79 AB + 7.25 AC }-\text{ 4.51 BC}\mathrm{+ 11.25 }{A}^{2}\mathrm{+ 9.70 }{B}^{2}\mathrm{+ 5.27 }{C}^{2}$$

**Fig. 1:**
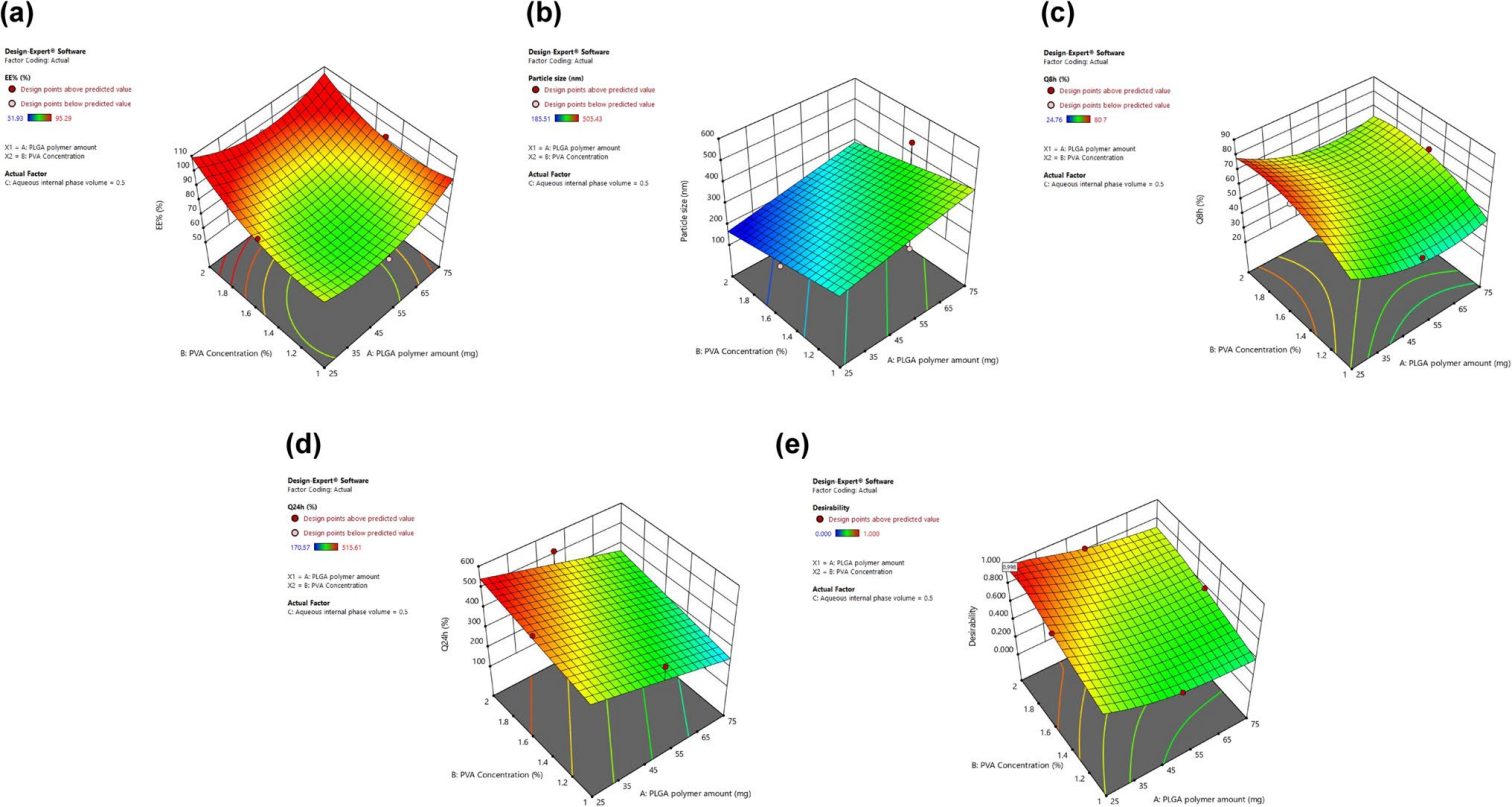
Response surface plots for the effect of amount of polymer PLGA (mg) (**A**), PVA concentration (%w/v) (**B**), and volume of aqueous internal phase (ml) (**C**) on (**a**) EE (%), (**b**) particle size (nm), (**c**) Q8h (%), (**d**) Q24h (Mcg/cm^2^), and (**e**) desirability of the developed DH-loaded PLGA NPs

The increased drug encapsulation at a higher concentration of the PLGA polymer, as illustrated in Fig. 1a, may be due to the increased viscosity of the organic phase. This prevents drug molecules from diffusing out to the aqueous phase during the homogenization step, resulting in enhanced drug entrapment within the NP matrix (Sharma et al. 2016). These results are in good agreement with those provided by Gao et al. (2006), who stated that the higher the amount of PLGA, the higher the viscosity, which resists the shear forces of the solution and inhibits drug leakage. Hence, it enhances encapsulation effectiveness.

#### Zeta sizer and potential particle size

Accompanied by particle size evaluation, the prepared PLGA NPs were shown to be in the nanoscale range. The size of the PLGA NPs generated was between 185.51 ± 15.14 and 505.43 ± 25.28 nm, as illustrated in Table 2. The PDI of all PLGA NPs was between 0.059 and 0.494, indicating a polydispersity system that did not exhibit a very narrow (PDI < 0.05) or very broad (PDI > 0.7) size distribution (Cho 2014). The ZP of all of the DH-PLGA NP formulations displayed a high negative value between −31.25 mV and − 75.36 mV, Supplemented as Table S1.

The linear model was determined to be suitable for the particle size data provided using ANOVA, and the adequacy/precision ratio of 9.889 suggests that the signal is sufficient. Equation (4) indicates the quantitative influence of the independent parameters on the particle size of DH–PLGA NPs in terms of coded values:

4 $$\text{Particle size }= 33920 + 63.52\, A- 59.19 \,B +49.19\, C$$

The results indicated that the size of DH-PLGA NPs was significantly influenced by the PLGA polymer (*p* < 0.05), as illustrated in supplemntary Table S2. Figure 1b illustrates that the organic phase’s resistance to flow increases as the quantity of PLGA polymer increases, while the organic phase volume remains constant. This results in a particle that is more consistent and requires a greater shear force to break.

### In vitro release study of DH-loaded PLGA NPS

The sink condition at 32 °C is used to evaluate the accumulative release behaviour of control DH and DH–PLGA NPs in SNES pH 5.5. DH–PLGA NPs gradually diffused into the release medium where the Q8 among various preparations ranged from 24.76 ± 5.22% to 80.70 ± 16.32%, in contrast to a much higher rate of pure DH at 3 h indicating dialysability, *p* > 0.05.

Despite the fact that the quadratic correlation between the in vitro release and the three independent factors was demonstrated with an adequate precision value of 11.204, predicted *R*^2^ (0.3861), and adjusted *R*^2^ (0.8848), only the PVA concentration had a significant impact on the tracked response. The PLGA polymer and aqueous internal phase volume did not have a significant impact, with *p* values of 0.0696 and 0.0620, respectively. Equation (5) demonstrates the quantitative influence of independent factors on the release of PLGA NPs in the form of coded values.

$$\text{Q8h = 46.12 - 4.68 A +12.34 B + 4.87 C }-\text{ 0.1100 AB + 2.61 AC + 3.81 BC}$$

5 $$\mathrm{+ 10.21 }{\mathrm{A}}^{2} \, -\mathrm{ 8.99 }{\mathrm{B}}^{2}\mathrm{ + 19.74 }{\mathrm{C}}^{2}$$

According to zero-order, first-order, and Higuchi equations, the release data were modeled. The pattern of DH release from the majority of PLGA NPs’ formulation matched the Higuchi equation, although certain PLGA NPs were fitted to zero- or first-order equations.

#### Ex vivo permeability study

In an effort to anticipate the in vivo enactment of the assembled PLGA NPs for intranasal delivery, ex vivo permeation studies were deconstructed. The cumulative quantity of DH was higher in the nasal mucosa, where DH-PLGA NPs were more permeated from PLGA NPs, which ranged from 170.57 ± 10.36 to 515.61 ± 29.41 µg/cm^2^ compared with the significantly slower permeation of 162.90 ± 14.0 µg/cm^2^ for crude DH, *p* < *0.05*, as demonstrated at Table 4. Collectively, Table 5 summarizes the computed permeation parameters of the examined DH-loaded PLGA NPs through nasal mucosa. The transdermal flux values for the investigated PLGA NPs ranged from 15.03 ± 0.8 to 55.16 ± 3.6 µg/cm^2^ h, compared to 9.77 ± 0.9 µg/cm^2^ h for the control DH solution.

**Table 4:** Ex vivo permeation parameters of DH-loaded PLGA NPs versus DH solution

Formulation	Lag time (min)	Jss (µg/cm^2^ h)	Kp (cm/h)	EI
P 1	24.94 ± 3.52	54.15 ± 2.84	0.0541 ± 0.0014	5.54
P 2	80. 69 ± 4.23	22.49 ± 0.86	0.0225 ± 0.0016	2.30
P 3	83.02 ± 5.46	23.52 ± 3.42	0.0235 ± 0.0017	2.41
P 4	51.05 ± 2.33	44.06 ± 0.99	0.0441 ± 0.0007	4.51
P 5	69.31 ± 4.66	32.74 ± 5.23	0.0327 ± 0.0013	3.35
P 6	93.62 ± 6.14	15.03 ± 3.87	0.0150 ± 0.0011	1.54
P 7	66.52 ± 10.51	26.54 ± 4.75	0.0265 ± 0.0008	2.72
P 8	38.53 ± 2.73	55.16 ± 6.12	0.0552 ± 0.0015	5.65
P 9	50.50 ± 9.81	40.66 ± 1.96	0.0407 ± 0.0027	4.16
P 10	74.27 ± 8.74	27.60 ± 4.57	0.0276 ± 0.0019	2.82
P 11	90.38 ± 11.45	19.30 ± 2.26	0.0193 ± 0.0016	1.98
P 12	60.39 ± 5.77	38.63 ± 5.94	0.0386 ± 0.0040	3.95
P 13	47.87 ± 6..01	41.79 ± 6.12	0.0418 ± 0.0031	4.28
P 14	79.65 ± 7.29	23.44 ± 3.43	0.0234 ± 0.0054	2.40
P 15	72.31 ± 8.74	.178 ± 30.11	0.0301 ± 0.0035	3.08
DH solution	100.20 ± 22.66	9.77 ± 2.51	0.0098 ± 0.0009	–

Data are mean values (*n*** = **3) ± SD.*Jss*, drug flux; *Kp*, permeability coefficient; *EI*, enhancement index

**Table 5:** Composition, actual, and predicted responses of the optimal DH-loaded PLGA NPs formulation

Factor	Optimal value	Response v ariable	Actual value	Predicted value	% Prediction error^a^
**A:amount of PLGA polymer (mg)**	**25**	**EE%**	**98.04 ± 3.71**	**99.87**	** − 1.87**
**B:conc PVA (%w/v)**	**1.85**	**Particle size** **(nm)**	**174.82 ± 11.45**	**185.51**	** − 6.11**
**C:volume of aqueous internal phase (ml)**	**0.5**	**Q**_**8h**_ **(%)**	**78.61 ± 5.33**	**80.16**	** − 1.94**
		**Q**_**24h**_ **(%)**	**515.69 ± 16.39**	**522.31**	** − 1.28**

*PLGA* poly lactide co-glycolide, *PVA* polyvinyl alcohol. ^a^Calculated as [actual – predicted/actual] × 100

Thus, the results obtained underscored the substantial function of PLGA NPs in the maintenance of DH emission and the enhancement of its nasal mucosa passage from 1.54 to 5.65 times that of the DH solution. The proposed sequential model for predicting the Q24 response was determined to be 3 FI with an adjusted *R*^2^ value of 0.6318, as revealed by ANOVA. This suggests that the model could account for nearly 63% of the total variance in the transdermal Q24. The Q24 may be associated with the three components in terms of coded value by employing the subsequent equation:

6 $$\mathrm{Q}\left(24\mathrm{h}\right)=318.12-87.25A + 60.19B- 72.28C$$

The Q24 of DH-loaded PLGA NPs is influenced by the formulation variables, as illustrated in Fig. 1d. The three independent variables significantly influenced the Q24 parameter (*p* < *0.05)*.

It has been observed that the concentration of PVA (B) has a positive regression coefficient, whereas the amount of PLGA polymer (A) and the volume of aqueous internal phase (C) have negative regression coefficients. This suggests that the transdermal diffusion is positively influenced by the decrease and increase in, respectively, their levels.

The permeability coefficient, permeated drug amount, lag time of formulation P1 (containing 50 mg PLGA, 2% (w/v) PVA, and 0.5 ml aqueous internal phase) were the highest and shortest, respectively. These findings can be attributed to the fact that P1 nanoparticles have the lowest aggregation and the highest surface area, which are a result of their small size and low polydispersity index. In addition, the occlusive effect is exacerbated by smaller particles, resulting in the most extensive occlusion with the tiniest particles. This has the potential to result in a significant increase in diffusion. The formulation (P6 with 50 mg PLGA, 1% (w/v) PVA and 1 ml aqueous interior phase) that had the lowest permeation parameters was determined by the most significant factor, which was the larger particle size. These discoveries correspond with those detected in scientific journals that have been published (Wissing and Müller 2002).

### Selection of the optimal PLGA NPs

The Design–Expert® software assessed the feasibility of optimizing the investigated responses in accordance with the obtained results. It was deemed desirable to predict the optimal formula with maximizing EE%, Q8h, Q24h, and minimizing particle size.

The predicted optimal DH-loaded PLGA NPs were identified, fabricated in triplicate, and evaluated for their effects. As depicted in Fig. 1e, the greatest value of desirability was found to be 0.998. This outcome desirability value suggested that the PLGA NPs composition contained 25 mg PLGA polymer, 1.85% w/v PVA, and 0.5 ml aqueous internal phase volume. The optimized formulation has an EE% of 98.04 ± 3.71% with a particle size of 174.82 ± 11.45 nm. The % release over 8 h and the amount of DH permeated through mucosa over 24 h from the optimized formulation were 78.61 ± 5.33% and 515.69 ± 16.39 µg/cm^2^, respectively. Table 5 illustrates that the optimized formula’s observed values were highly similar to those predicted, with a negligible prediction error that ranged from 1.28 to 6.11% for a variety of responses. This highlights the sufficiency and appropriateness of the proposed mathematical model for the prediction of dependent responses. Consequently, this preparation was employed in the subsequent investigations.

### Particle morphology

**Fig. 2:**
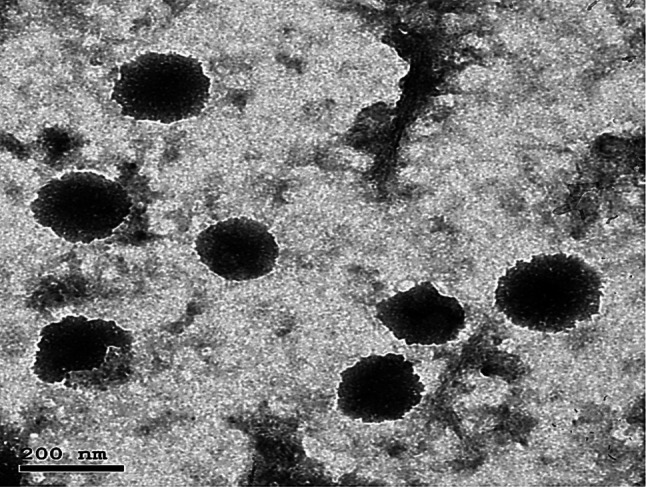
Transmission electron micrograph of the optimal DH-loaded PLGA NPs formulation

The morphology of the optimal PLGA NPs is shown in Fig. 2. The NPs were found to be distinct, spherical, solid, and dense assembly structures with a small PDI. Morphology analysis confirmed the previous particle size estimates determined using DLS.

### Stability study of PLGA NPs

**Fig. 3:**
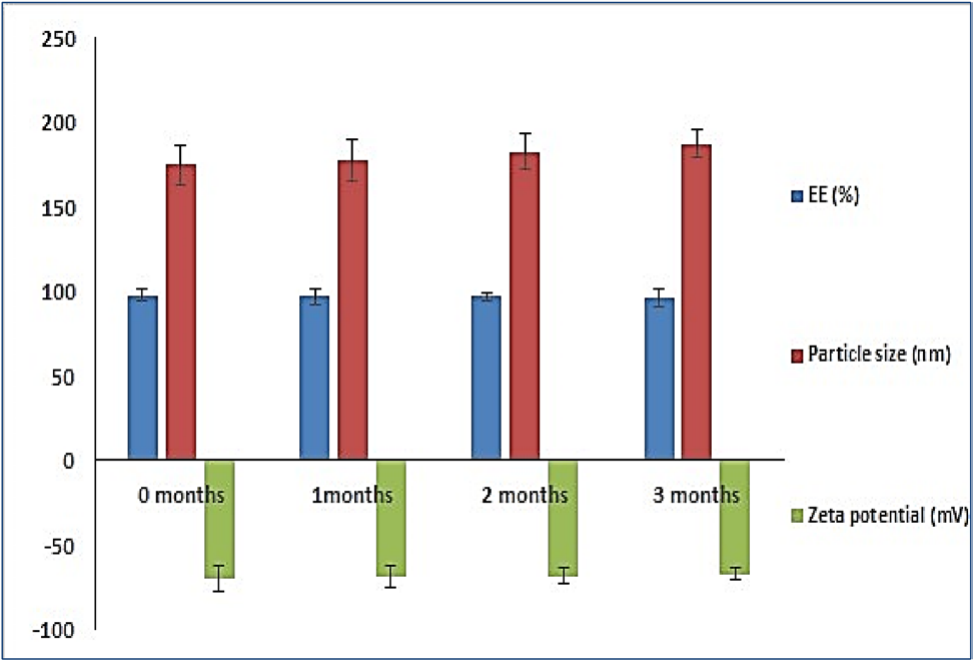
Effect of storage on EE%, particle size and zeta potential of DH–PLGA NPs

After 3 months of storage at 4 °C, the stability of the enhanced PLGA NPS formulation was examined. The ideal formulation of PLGA NPS exhibited excellent physical stability without discernible alteration in appearance. The DH EE%, size, zeta, and PDI did not vary significantly (*p* > *0.05*) after storing for 3 months, showing the kinetic stability of the stored PLGA NPS over the period (Fig. 3). This exceptional storage stability may be attributable to the optimally tiny particle size and narrow PDI.

### Differential scanning calorimetry (DSC)

**Fig. 4:**
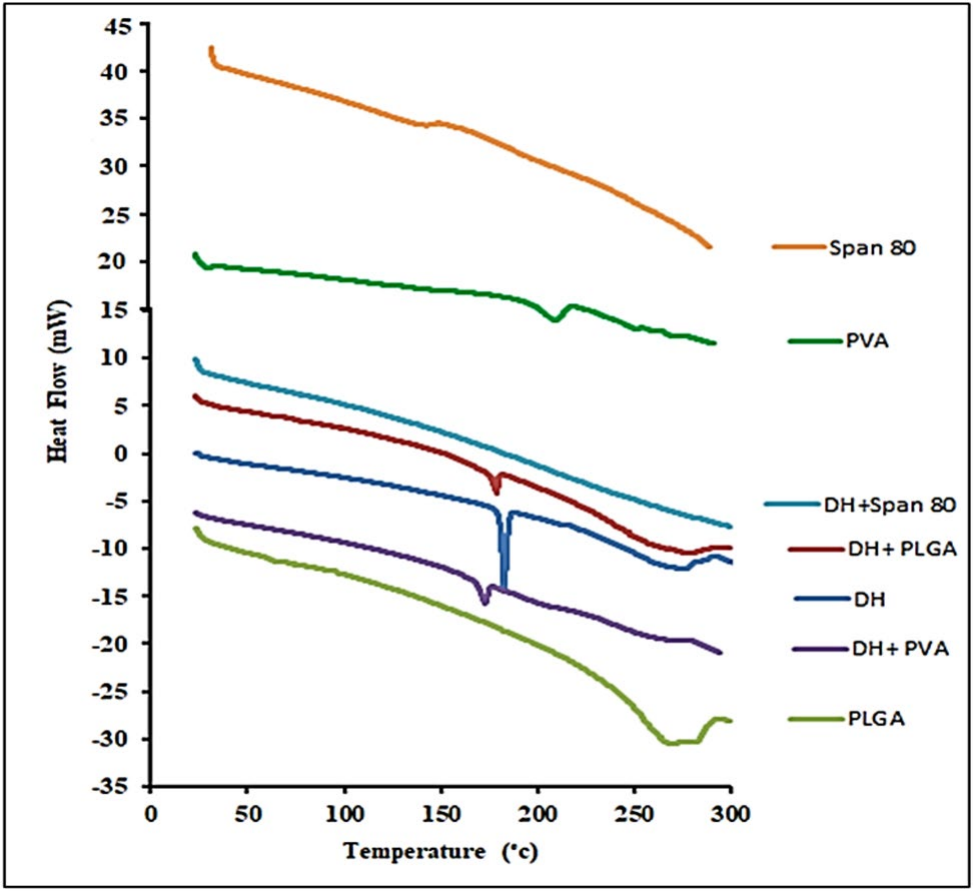
DSC thermograms of DH, PLGA, PVA, span 80, DH–PLGA binary mixture, DH–PVA binary mixture, and DH–span 80 binary mixture

The presence of a potential physicochemical interaction between DH, PLGA, PVA, and span 80 was investigated using DSC. The th.ermograms of DSC data are illustrated graphically in Fig. 4.

The thermogram of DH exhibited a single primary melting endothermic peak at 182.31 °C. Endothermic peaks are observed in the DSC thermograms of Span 80 at − 12 ℃ (Kato et al. 2008). The physical mixtures of DH–Span 80 show that the peak for Span 80 was not observed in the DSC because the instrument did not run below 0 ℃, but not of the drug. The DSC thermogram of PLGA and PVA shows that PLGA and PVA alone exhibit an endothermic peak at 67.04 °C (Mainardes and Evangelista 2005) and 217.13 °C (Asran et al. 2010), respectively. The endothermic peak of the physical mixture of DH–PLGA and DH–PVA was observed at the position corresponding to the melting point of DH (180.82 °C) and (180.62 °C), respectively. However, the peak corresponding to the melting point of PLGA and PVA vanished, which could be attributed to the plasticizing effect of residual water (Guo et al. 2015).

### In vivo study in yohimbine- induced premature ejaculation in normal and diabetic rats

#### Ejaculation frequency

**Fig. 5:**
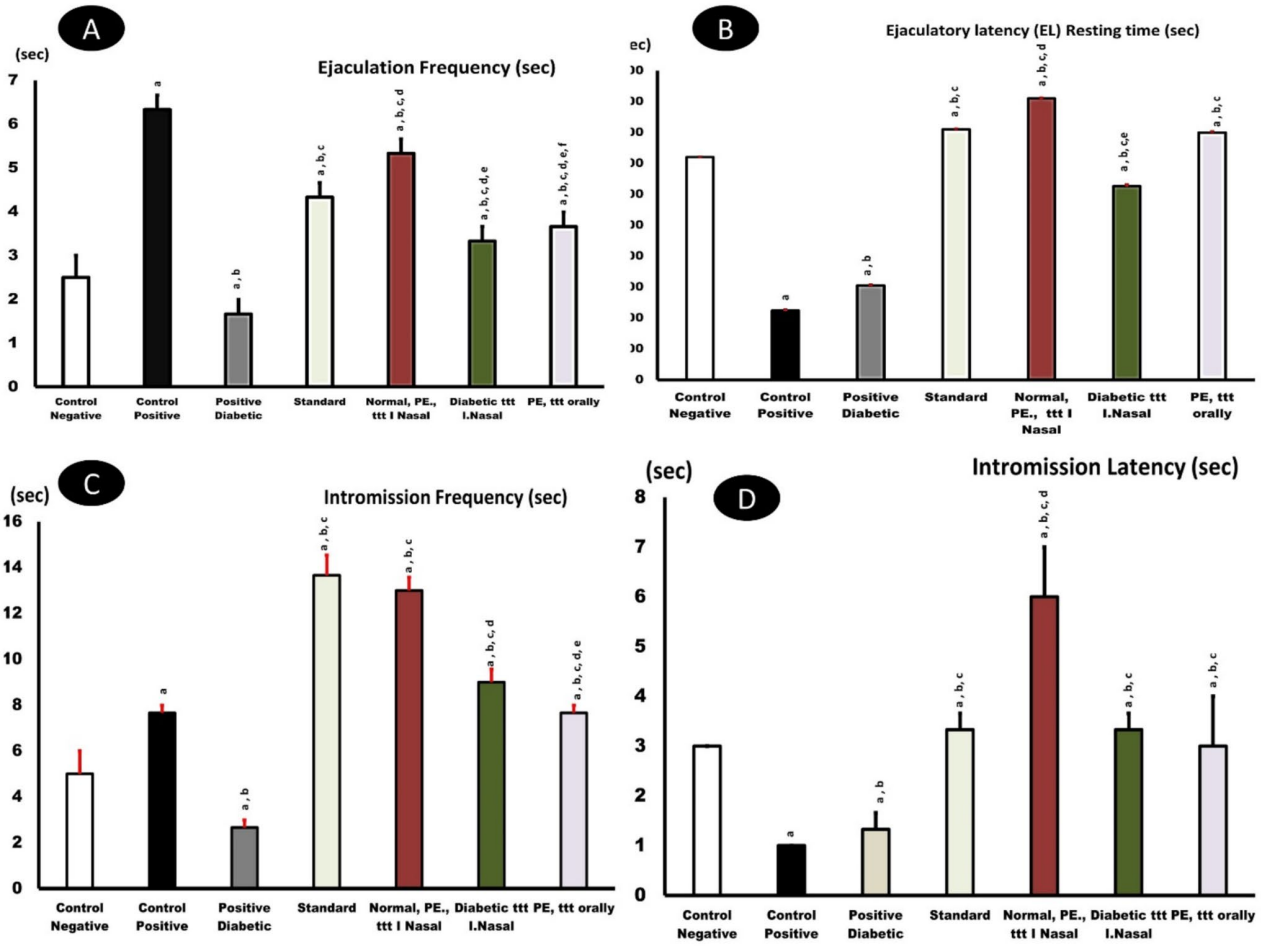
Effects of dapoxetine in normal (orally administered) or IN nano form in normal and diabetic rats in comparison with control negative (normal), control positive (PE), and standard treated rats (Lidocaine) on Ejaculation frequency (EF), Ejaculation latency (EL), Intromission frequency (IF), and Intromission latency (IL). ^a^*p* < *0.05* relative to the negative control; ^b^*p* < *0.05* relative to the positive control; ^c^*p* < *0.05* relative to the positive diabetic; ^d^*p* < *0.05* relative to the standard group; ^e^*p* < *0.05* relative to *PE normal*; ^f^*p* < *0.05* relative to *PE diabetic (diabetic ttt IN) and orally treated one*

The ejaculation rate or frequency of PE-induced rats that were administered dapoxetine formula IN exhibited a significant increase (*p* < *0.05*) in comparison to the control group, dapoxetine orally treated group, and diabetic patient, as illustrated in Fig. 5A.

Disease models (control positive and positive diabetic) showed PE: compared to the control negative group (normal animals), both the control positive (PE-induced) and positive diabetic (diabetic PE-induced) groups exhibited significant detrimental effects, confirming the successful induction of the disease model. Decreased latency: both groups showed a significant reduction in ejaculation latency (EL) (Fig. 5B) and intromission latency (IL) (Fig. 5D), indicating rapid ejaculation. Altered frequencies: The PE groups generally displayed a reduction in ejaculation frequency (EF) (Fig. 5A) and intromission frequency (IF) (Fig. 5C), suggesting overall lower sexual performance. Standard treatment efficacy: the standard group (likely treated with an established method) demonstrated significant improvement in all measured parameters compared to the untreated PE models. This serves as a positive mark. Superiority of IN nanogel treatment: the group that is diabetic treated with the IN (diabetic treated IN with the nanogel) showed the most pronounced therapeutic effect. Ejaculation latency (EL): The IN nanogel group demonstrated a significant increase in EL (Fig. 5B), approaching or even surpassing the normal and standard groups. Comparison to oral: In most parameters (Fig. 5A, B, C, D), the diabetic IN group performed better than the orally group, indicating that the intranasal route and/or the nanogel formulation enhanced the drug’s efficacy. Therapeutic benefit in PE and diabetic PE treatment with the drug (both standard and nanogel) effectively countered the behavioural deficits caused by both PE and the diabetic PE condition, indicating the formulation is potent even in the presence of complex diabetic pathology.

#### Ejaculation latency

PE treatment focuses on ejaculation latency (EL), the time between vaginal interaction and ejaculation. In both normal and diabetic patients, IN dapoxetine-treated rats had a longer duration than control patients or normal rats (Fig. 5B).

#### Intromission frequency

Intromission increases with medicinal effectiveness. The synthesized medication had a higher intromission frequency than the oral dapoxetine capsules in the same standard activity (*p* < *0.05*) (Fig. 5C**)**.

#### Intromission latency

Nano-synthesized medications given IN to normal and diabetic people exhibit powerful aphrodisiac effects. Figure 5D shows lower IL levels in diabetic and PE patients.

#### Mount frequency

**Fig. 6:**
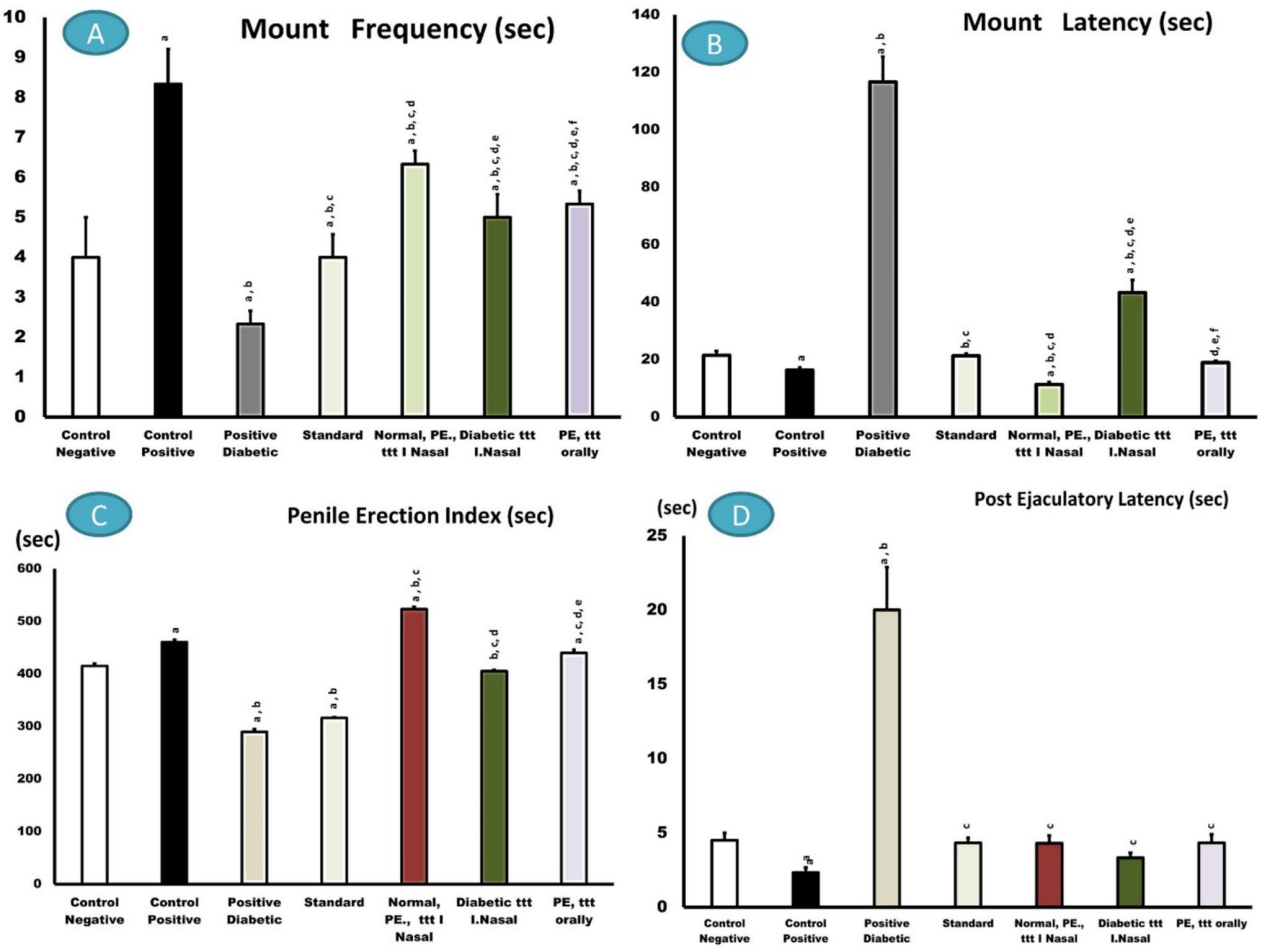
Effects of dapoxetine in normal (orally administered) or IN nanoformula in normal and diabetic rats in comparison with control negative (normal), control positive (PE), and standard treated rats (Lidocaine) on MF, ML, PEI, and PEL

In this study, a substantial increase (*p* < 0.05) in mount latency was observed in normal and diabetic rats that were administered dapoxetine IN, nanoformula, followed by oral conventional powdered DH not formulated, standard, and control negative groups. The diabetic patient’s mount frequency decreased (Fig. 6A).

#### Mount latency

Significant decrease in fresh mounting (*p*<*0.05*) or in the ML dapoxetine formula administered IN, which is quicker than the conventional drug. However, diabetic and PE patients and diabetic non-treated rats showed the opposite effect Fig. 6B.

#### Penile erection index

Dapoxetine-treated IN to rats, both normal and diabetic, had higher penile erection indexes. Standard and control rats received oral dapoxetine (Fig. 6C).

#### Post ejaculatory latency

As demonstrated in Fig. 6D, the time needed to ejaculate again depends on the PEL, which reduced significantly (*p* < *0.001*) in all groups compared to PE patients.

#### Ano-genital sniffing

**Fig. 7:**
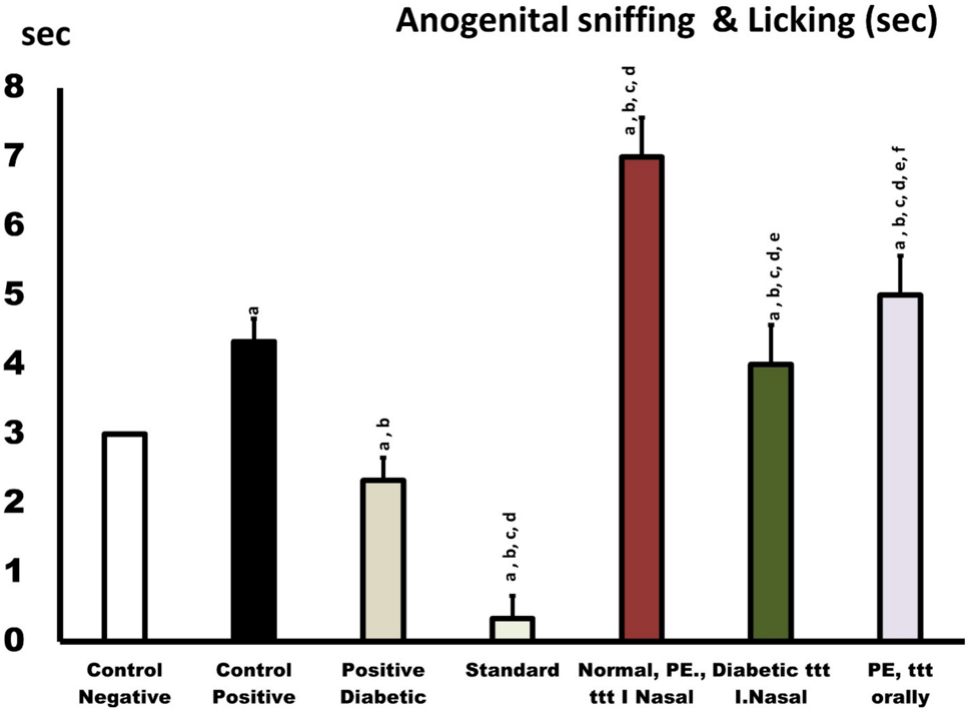
Effects of dapoxetine in normal (orally administered) or IN nano form in normal and diabetic rats in comparison with control negative (normal), control positive (PE), and standard treated rats (Lidocaine) on ano-genital sniffing and licking.^a^*p* < 0.05, relative to the negative control; ^b^*p* < 0.05, relative to the positive control; ^c^*p* < 0.05, relative to positive diabetic; ^d^*p* < 0.05, relative to standard group; ^e^*p* < 0.05, relative to PE normal; ^f^*p* < 0.05, relative to PE diabetic

In normal and diabetic individuals, in dapoxetine nano form increased ano-genital sniffing. However, in lidocaine-treated rats, ano-genital sniffing decreased significantly (*p*<0.01), confirming the standard spray’s negative effects on nasal mucous membrane sensitivity and other side effects (Fig. 7).

Supplemented Tables 3 and 4 (S3 and S4 tables) show all sexual activity characteristics of male rats with premature ejaculation in normal and diabetic patients added as supplementary data.

#### Olfactory region safety and drug toxicity

Normal intact nasal walls exhibited average epithelial lining, sub-mucosa, blood vessels, and cellularity in the intra nasal administered group, as well as nose cartilage, whereas other sites exhibited intact epithelial lining, sub-mucosal oedema, and somewhat congested blood vessels, supplemented as Fig. S1.

#### FOS protein immunohistochemistry

Acute duloxetine and FOS. Fast ejaculator diabetic rats (PE) treated with I/N dapoxetine had similar brain stem FOS expression to fast rats treated with vehicle. After dapoxetine immediate treatment, sexual categorization impacted more brain FOS areas. PE control positive rats and PE orally treated with dapoxetine had high FOS cell densities. Finally, IN dapoxetine nano-platform and lidocaine standard rats had the lowest FOS cell density (Table 6) and also in the supplmemted Fig. S2

**Table 6:** Effects of dapoxetine in oral and intranasal nano platform on the neurological activity and percentage of positive immune cells in different areas of the brain in premature (rapid ejaculator) male rats after ejaculation to estrus-induced female rats (mean ± SE)

Percentage of positive immune cells	Group
19.66 ± 1.45^b^	G1 (Control negative) (Normal)
79.33 ± 4.05^a^	G2 (Control positive) (PE)
15.33 ± 1.45^c^	G3 (Standard)
88.33 ± 2.02^a^	G4 (Positive diabetic)
6.66 ± 1.45^d^	G5 (PE, IN)
19.00 ± 4.72^b^	G6 (Diabetic, PE, IN)
72.33 ± 4.33^a^	G7 (PE, diabetic, orally)

*PE* premature ejaculation, *IN*intranasal. Values with different superscript letters revealed significant differences from each other at *p* < 0.001. It isobserved that a highly significant difference at *p* < 0.00 is observed in the percent of positive immune cells between the different study groups

### Histopathological studies

**Fig. 8:**
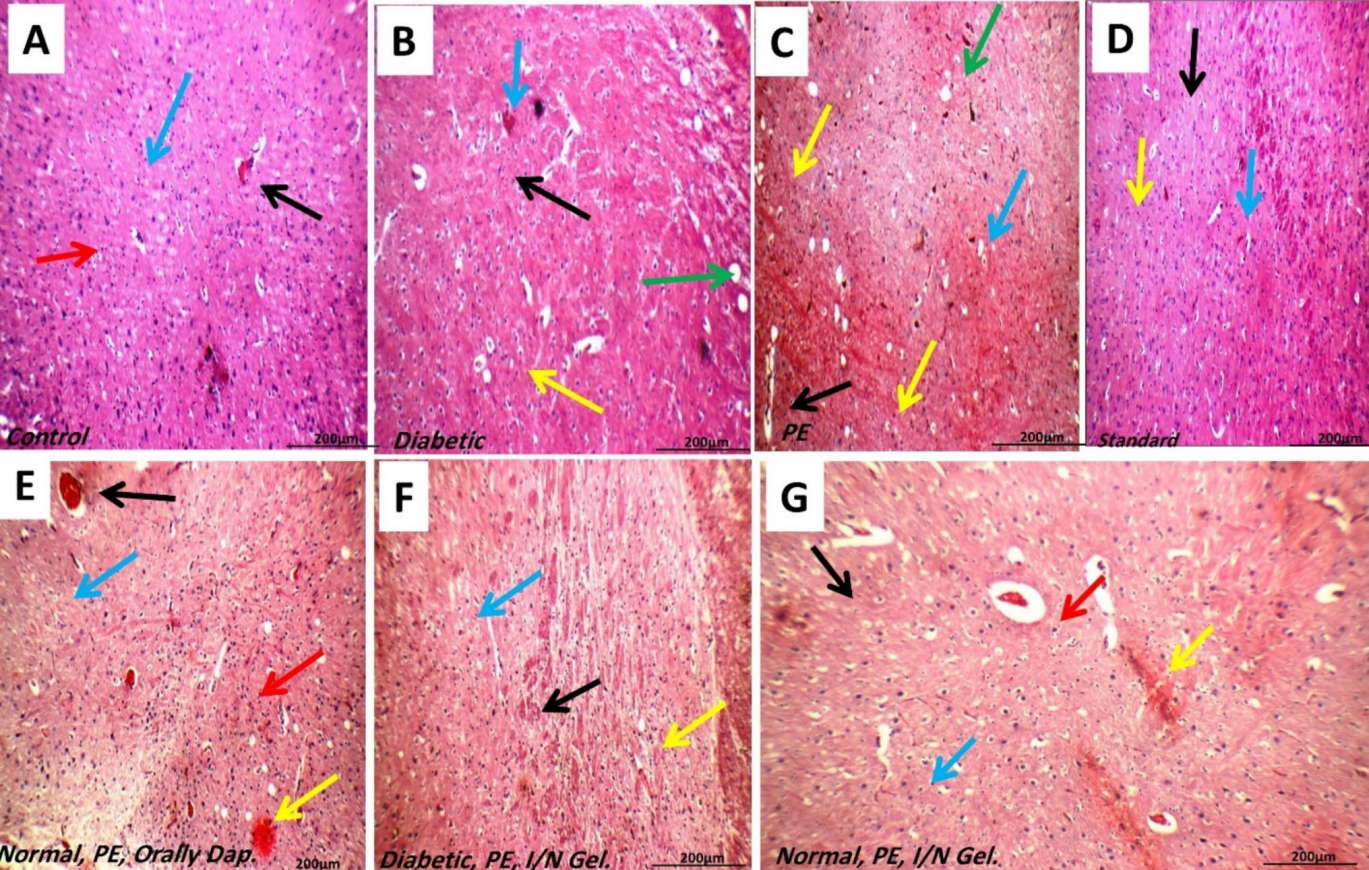
Brain histopathology. IN injection of dapoxetine in normal (**A**) and diabetic rats (**B**) compared to control positive (PE) (**C**), and standard treated rats (Lidocaine) (**D**) on ano-genital sniffing and licking compared to normal orally treated rats (**E** and **G**). PE shows distributed degraded glial cells in a fibrillary background with microcyst formation, congested intra-cerebral blood arteries, and deteriorated neurons with intra-cytoplasmic vacuoles. Intracerebral blood arteries with peri-vascular eosinophilic plaque-like patches, degenerative neurons, and glial cells are spread in a fibrillary background with modest microcyst formation in diabetic rats (**B**). In normal and diabetic rats, oral PE therapy caused modestly clogged blood vessels (black arrow), distributed damaged neurons, average glial cells, and eosinophilic plaque-like patches. However, IN therapy significantly decreased these results (**F** and **G**). The negative control brain has average intra-cerebral blood arteries (black arrow) and glial cells (blue arrow) in a fibrillary backdrop. **B** Diabetic brain: average intra-cerebral blood vessels (black arrow), peri-vascular eosinophilic plaque-like region (blue arrow), average glial cells (yellow arrow), and modest microcystformation. Premature group: brain with minimally clogged intra-cerebral blood arteries (black arrow), scattered degenerative neurons (blue arrow), glial cells (green arrow), and considerable microcyst development. **D** Normal treatment: brain with average intra-cerebral blood arteries (black arrow), dispersed degenerative neurons (blue arrow), and average glial cells. **E** Premature intranasal nano gel-treated brain demonstrating severely congested intra-cerebral blood arteries (black arrow), average glial cells (blue arrow), eosinophilic plaque-like regions (yellow arrow), and average neurons (red arrow). **F** Diabetic treated with intranasal nano gel: brain exhibiting average neurons (black arrow), glial cells (blue arrow), and neurons. **G** Normal, non-diabetic premature-IN-treated brain with average neurons (black arrow), glial cells (blue arrow), modestly congested blood vessels (red arrow), and eosinophilic plaque-like regions (H&E × 200)

After acute administration with normal or nano dapoxetine in IN or orally administered rats, along with control positive and negative groups, brain histological examination included cerebral and hippocampal region studies. Control group brains had average intra-cerebral blood vessels, neurons, and glial cells in the fibrillary background. After normal treatment, prematurely IN nano formula-treated rats had average intra-cerebral blood vessels, scattered deteriorated neurons, degraded glial cells on a fibrillary background, and a few scattered micro cysts. Average intra-cerebral blood arteries with peri-vascular eosinophilic plaque-like patches, scattered deteriorated neurons, and scattered degraded glial cells in a fibrillary background with modest micro cyst formation were seen in diabetic rats; all problems improved following intranasal nano gel treatment. Oral dapoxetine treatment in PE rats showed modestly clogged blood arteries, distributed degraded neurons, average glial cells, and eosinophilic plaque-like areas. After IN treatment, all of these traits were significantly decreased (Figure 8).

In the control group, average Cornu Amonis (CA1), (CA2), (CA3), dentate gyrus (DG), pyramidal neurons, granule cells, inter-neuron area, and hippocampus blood vessels. In CA1 and DG, pe-treated mice displayed scattered pyramidal neurons and granule cells, dispersed neurons with vacuolated cytoplasm in CA3, oedematous inter-neuron region, and moderately congested blood vessels. The rats had average pyramidal neurons and granule cells in Cornu Amonis (CA1), (CA2), and (CA3) after conventional lidocaine spray therapy, dispersed degenerated pyramidal neurons with average DG, somewhat oedematous inter-neuron area, and average blood vessels.

**Fig. 9:**
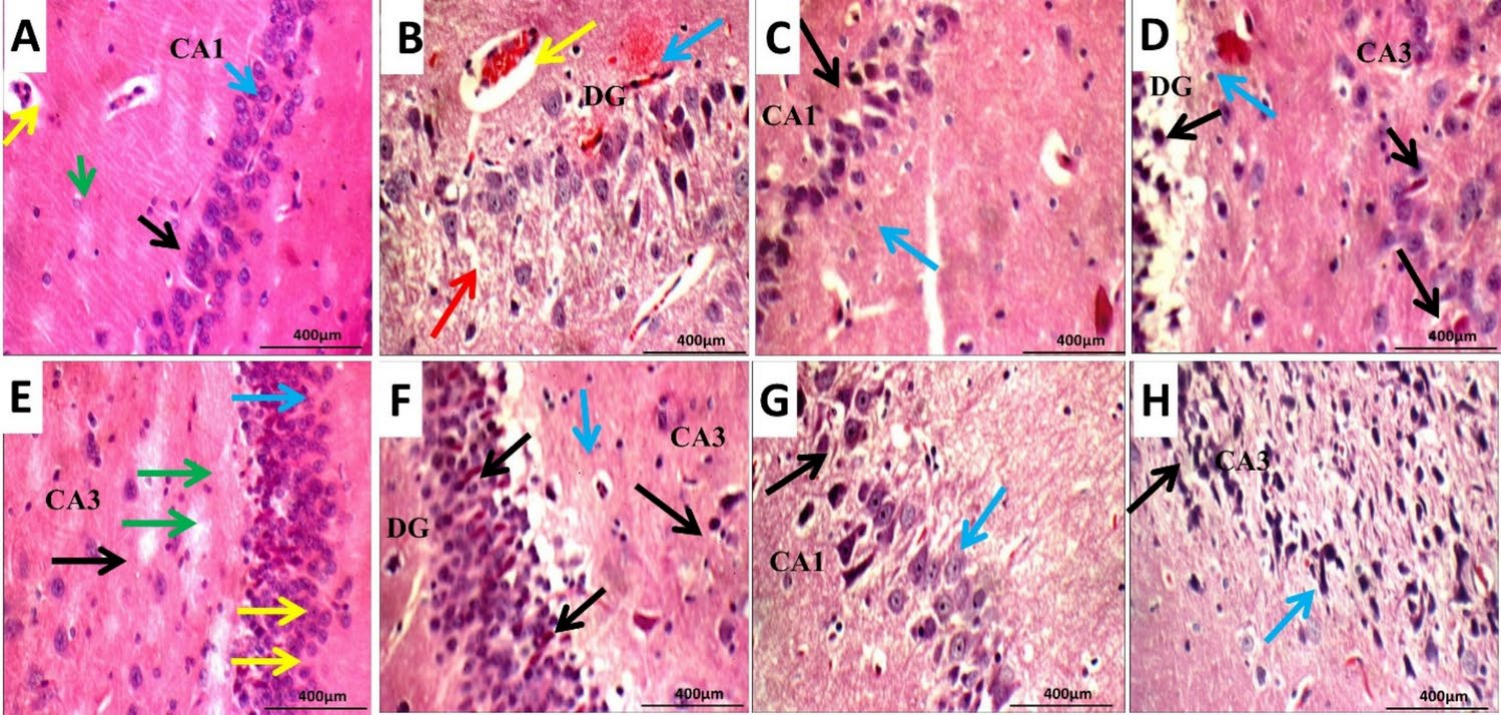
Hippocampus histopathology: the hippocampus of diabetic non-treated rats (**B**) and PE-induced rats (**C**) had a distinct Cornu Amonis (CA1) with scattered degenerated pyramidal neurons (black arrow) and granule cells with inter-neuron blood vessel congestion and peri-vascular eosinophilic plaque-like areas (CA3). The conventional treatment group (**D**) had significantly fewer deteriorated pyramidal neurons (black arrow). Premature oral dapoxetine (**E**) treatment caused an unclear Cornu Amonis (CA1) with distributed degenerative neurons, a slightly oedematous inter-neuron region, and an eosinophilic plaque-like area in the hippocampus. In contrast to the PE, diabetic rodents (**F**) and PR normal group (**G**), the IN-treated diabetic or normal rats did not develop degenerative alterations. (H&E × 400). **C** negative: higher power image of average pyramidal neurons (black arrow) and granule cells (blue arrow) in CA1, average inter-neuron region (green arrow), and slightly congested blood arteries. **B** Intranasal nano gel-treated premature: average neurons (red arrow), congested blood vessels (yellow arrow), and dispersed neurons (blue arrow). **C** Diabetic treated with intranasal nano gel: higher power picture of distributed degenerated pyramidal neurons (black arrow) in CA1 and average inter-neuron area (blue arrow). **D** Intranasal nano gel-treated diabetic: another image shows scattered deteriorated pyramidal neurons (black arrow) in (CA3) and (DG) and moderately oedematous inter-neuron region (green arrow) in (DG). **E** Standard treatment: scattered degenerating pyramidal neurons (yellow arrow) with average DG granule cells (blue arrow), average CA3 neurons (black arrow), and moderately oedematous inter-neuron region. **F** Diabetic treated by intranasal nano gel: another view showing scattered degenerated pyramidal neurons (black arrow) in (CA3) and (DG), and mildly oedematous inter-neuron area (green arrow) in (DG). Premature treated: higher power view showing scattered. (H&E × 400)

Diabetes-untreated rats had distinct Cornu Amonis (CA1) with scattered degenerated pyramidal neurons and granule cells, indistinct (CA3) with markedly degenerated pyramidal neurons and oedematous inter–neuron area, and distinct dentate gyrus (DG) with scattered degenerated pyramidal neurons. Diabetes treated with IN Gel exhibited deteriorated pyramidal neurons in the Cornu Amonis (CA1), CA2, CA3, and DG, mildly oedematous inter-neuron region, and modestly congested blood vessels. PE treated with IN Gel had distinct Cornu Amonis (CA1), (CA2), (CA3), and dentate gyrus (DG), but some degenerative neurons in CA1, eosinophilic plaque-like regions in CA3 and DG, moderately oedematous inter-neuron areas, and severely congested blood vessels in DG. Amonis (CA1) had scattered degenerated neurons, mildly oedematous inter-neuron area, and eosinophilic plaque-like area in prematurely treated hippocampus with oral dapoxetine. CA 2 and CA 3 had scattered degenerated neurons and plaque-like area, while DG had average neurons and mild oedema (Figure 9).

## Discussion

There are several psychosexual and pharmaceutical treatments for premature ejaculation. Psychosexual psychotherapy and daily or on-demand medication are used alone or in combination to treat PE. Lifelong premature ejaculation (L-PE) in males is best managed with PE medication alone or in combination with patient and couple psychosexual treatment. Men with A-PE should get etiology-specific treatment, such as psychosexual psychotherapy or ED medication, alone or in combination with PE. Psychosexual education, graded patient, and couple psychotherapy are best for males with natural variable ejaculatory dysfunction (PE) or PE-like symptoms (Althof et al. 2014).

A variety of pharmacotherapeutic approaches have been implemented to manage PE (Giuliano and Clèment 2012). Interventions include alpha adrenergic blockers, topical local anaesthetics (LA), PDE5i, tramadol, and SSRIs. The first known pharmacological therapy for PE was topical LA like lidocaine, procaine, or benzocaine to lower glans penis sensitivity (Schapiro 1943). However, in our investigation, we tried through nanotechnology to increase the efficacy with rapid acting and sustained control while targeting the brain centres for rapid effect through the IN administrations.

DH nanoformulation, depending on the amount of PLGA employed, increasing PVA levels showed varying impacts on EE%. A substantial increase in EE% was observed at a PVA concentration of 25 mg of PLGA. This may be attributed to the increased viscosity of the continuous phase as the concentration of PVA increases. This, in turn, impedes the diffusion of DH from the internal to the exterior aqueous phase, resulting in a greater drug encapsulating capacity (Zambaux et al. 1998). Moreover, PVA, a polymer that dissolves in water and has a high level of biocompatibility and biodegradability (Abdelkader et al. 2016), was chosen and added to the exterior aqueous phase during the fabrication of NPs to make them more stable and easy to redisperse (Zweers et al. 2003). At 75 mg of PLGA, EE% began to decline significantly as PVA concentration increased. As a hydrophilic medication, DH is encapsulated within the PLGA NPs (1ry emulsion) and PVA alleviates the NP by dispersing the H_2_O molecules to create a coacervate rich in polymers (Sharma et al. 2014). Squeezing the nanoparticles enhanced the drug’s solubility in water at higher concentrations of PLGA (75 mg), which increased drug partitioning to the exterior aqueous phase and reduced drug trapping inside the polymeric vesicle (Seju et al. 2011).

It was found that the EE% is significantly influenced by the volume of the aqueous internal phase. EE% decreased as a result of the increase in the quantity of the aqueous internal phase. The drug’s propensity to escape from the polymeric matrix and into the exterior aqueous phase was increased by the increased internal aqueous phase volume, which could potentially reduce drug entrapment. (Narayanan et al. 2014). According to Li et al. (1999), the primary emulsion is created by the thickness of the organic phase that surrounds the drug’s aqueous solution, which is a significant factor in the entrapment efficiency. Increasing the volume of the internal aqueous phase may result in the organic phase loosening and drug molecules escaping to the exterior aqueous phase to proliferate. The PDI was employed to examine the total homogeneity of the particulate size within the PLGA NPs and the diameter of the PS distribution. A heterogeneous distribution of the vesicles is indicated by a high PDI value, whereas a low PDI value indicates a homogeneous, monodispersed size distribution (Cho 2014). This range of ZP values typically indicates that the nanoparticles are highly dispersed in the aqueous medium and that the nano suspensions will exhibit exceptional stability and tolerance to aggregation.

This leads to the formation of larger oil droplets with a significant increase in particle size (Abou-Taleb et al. 2024). Beck–Broichsitter et al. (2010) Similarly, the results were reported, indicating that the viscosity of the organic phase increases, which leads to a delayed diffusion of solvent into the external phase and a greater aggregation of polymers. In contrast, the particle size of the DH–PLGA NPs that were fabricated was significantly reduced by the PVA concentration. Initially, the NPs’ size decreased as a result of the increased PVA level, which implies that PVA prevents oil particle coalescence and maintains the stability of the w1/o/w2 emulsion. Additionally, the decreasing tendency of NPs’ size with the subsequent increase in PVA level may be attributed to the reduced interfacial tension between the aqueous and organic phases, which results in smaller globules and a decrease in the PS of the NPs (Feng and Huang 2001). These results are comparable to those published by earlier investigations (Zweers et al. 2003; Allémann et al. 1992; Quintanar-Guerrero et al. 1996). But they disagree with other studies (Abdelkader et al. 2016).

The size of NPs was unfavorably affected by the positive effect of internal aqueous phase volume (C) when low and medium amounts of PLGA were used. The larger volume of internal aqueous phase during the second emulsification step of PLGA NP preparation led to an inefficient homogenization, an unstable w/o emulsion, and an increase in the frequency of collisions. These findings may suggest a significant increase in size (Li et al. 1999). In contrast, the use of a high amount of PLGA resulted in a significant decrease in the NP size, which may be attributed to the increased viscosity of the primary emulsion. This could potentially counteract the resistance of the viscous emulsion to the shear stress produced by homogenization (Crotts and Park 1995). To elucidate the primary theories for drug release from these NPs, the primary hypotheses have been the degradation of the polymer matrix and the diffusion of drug molecules (Zhou et al. 2001). Wu et al. (2005) reported that drug molecules located close to a nanoparticle’s surface were easily capable of diffusing from the NP matrix to the diffusion milieu during the preliminary incubation time. According to the release outline achieved for DH–PLGA NPs, the majority of the DH was located a considerable distance away from the surface of the nanoparticles. Moreover, degradation of the polymers slowed DH’s release (Jafarinejad et al. 2012).

Higher DH release was obtained in the formulations containing a lower amount of PLGA polymer. This may be due to decreased viscosity of the dispersion at a lower amount of polymer, which in turn would not retard the drug release (Illum et al. 2001). Additionally, the drug release would be facilitated by the high flexibility of the polymer backbone structure at lower concentrations. In addition, the reduced drug release may be attributed to the rapid solidification of the polymer-rich phase, which was a consequence of the increased polymer content in the first emulsion. This resulted in a dense matrix with low permeability to the encapsulated drug and a more contorted structure as a result of chain entanglement (Yang et al. 2001). In addition, the in vitro drug release of DH-PLGA NPs was more rapid at a lower concentration of PLGA than at a higher concentration. A larger surface area or a brief distance from the nucleus of the nanoparticle may be the cause of this phenomenon (Makadia and Siegel 2011).

Concurrently, the nanoparticles’ enhanced DH release rate was associated with the increase in PVA concentration. This may be due to the fact that the nanoparticles are of a minute dimension and contain a 2% concentration of PVA, which adds to the release medium’s surface area/volume ratio. These results and findings are corroborated by Cui et al. who asserted that the drug release rates of NPs with a higher concentration of PVA in the external phase were substantially accelerated (*p* value < 0.05) in comparison to those with a lower concentration (Cui et al. 2005). It had a positive and favorable effect on the release rate of PLGA NPs in relation to the aqueous internal phase volume. This observation could indicate the presence of a discrete PLGA NP with evident surface pores, which would generate a particulate matrix that is responsive to the entrance of the release medium and the dissolution of the payload (Crotts and Park 1995).

The enhanced nasal permeation that the prepared PLGA NPs have achieved may be attributed to the lipophilic nature, small particle size, and formation of a monolayer film, which establishes intimate drug contact with the mucous membrane. This results in a large surface area. The occlusive characteristics of this monolayer coating, which are hydrophobic, prevent drug loss of efficacy due to evaporation, hence facilitating drug penetration (Wissing and Müller 2003). In addition, the loss of water from the PLGA NPs produces crystal alteration of the PLGA NPs’ matrix, which can facilitate drug ejection and penetration (Borgia et al. 2005; Teeranachaideekul et al. 2008).

Men who are socially awkward often have premature ejaculation. Dapoxetine is the best therapy for this sexual disease; however, because ejaculatory delay and social anxiety are associated, it must be treated quickly. Rapid ejaculator rats were chosen from sexually trained rats after yohimbine doses caused premature ejaculation. Dapoxetine was tested as a nasal spray for rats with fast ejaculation. IN spray improved this condition and delayed intra-vaginal ejaculation compared to a saline control after treatment and before exposure to receptive females (Gengo et al. 2005). Rats given IN dapoxetine Nano platform had the longest ejaculatory latency, followed by oral and traditional rats. This study measured sexual parameters (Figs. 5, 6, and 7 and Table 5) and found that the IN nano platform increased intra-vaginal ejaculation latency by 4 min.

Oral dapoxetine tablets treat acquired or chronic PE. Though it delays fast ejaculation, it must be digested for at least two hours before sexual activity. Besides IN dosage, which has never been studied, this project aims to produce a nano formulation of dapoxetine to boost efficacy and provide quick effects before sexual activity. IN administration, fast brain targeting, and long-lasting effects, particularly for men with limited sexual experiences who require on-demand medication or prefer regular prescriptions. Drug penetration into the brain directly via the nose tube and BBB bridge is possible due to the large vasculature in the olfactory pathway and nasal epithelium (Illum 2004).

For ages, yohimbine has been used as an aphrodisiac and adrenergic 2 receptor blocker. Pharmacology texts claim it is an unproven aphrodisiac. Due to its role in noradrenaline autoregulation, inhibiting two boosted secretion and levels, which improve sexual function. Yohimbine improved erectile dysfunction in older male rats, according to studies. (Smith and Davidson 1990). Yohimbine was employed for fast ejaculation induction in rats in this investigation.

The discipline of drug delivery technology values non-invasive trans mucosal nasal medicament administration. The high vascularity, large surface area, avoidance of hepatic first-pass metabolism, gut wall metabolism, and/or gastrointestinal tract degradation explain this. Due to these factors, the IN route was the most effective for quick drug transport into the brain, delaying induced rapid ejaculator or premature rats, indicating that the medication can be delivered immediately before sexual activity, unlike the dapoxetine oral admixture. It may be against BBB’s nature to convey drugs. (Illum 2004). Consequently, many strong medications are rendered useless owing to insufficient brain drug delivery (Misra et al. 2003). Despite great attempts, conventional administration has only limitedly improved brain medication absorption. The intranasal cavity absorbs medications better and faster than other routes due to its structure, rapid blood flow, and large surface area. IN administration was confirmed by nasal mucosa histopathology showing no discomfort.

Intranasal administration significantly enhanced the brain bioavailability of DNP liposomes compared to conventional dosage forms and administration modalities. A nasal delivery of a liposomal formulation of a CNS medication can effectively circumvent the blood–brain barrier and minimize the risk of drug leakage into non-target tissues. The intranasal liposomal formulation delivery offers rapid distribution to the central nervous system, bypasses hepatic first-pass metabolism, eliminates the necessity for systemic treatment, and reduces undesirable systemic adverse effects. This approach’s sustained-release characteristic allows for self-administration by patients without significant distress or difficulty, potentially reducing the frequency of administration.

Pattij and colleagues (Pattij et al. 2005b) showed that FOS immunoreactivity increased from sluggish to active male rats in brain areas preferentially activated by ejaculation. FOS immunoreactivity was higher in most brain areas specifically triggered by ejaculation in sluggish male rats than active male rats (Coolen 2005). The present FOS expression study confirms these prior results that neuronal activity in certain hypothalamus and thalamic substructures is linked to ejaculatory behaviour. Normal rats’ cerebral cortex or hippocampus had normal histological structure and normal cerebral blood vessels, unlike diabetic or rapid ejaculator rats, which had congested intra-cerebral blood vessels, scattered deteriorated neurons with intra-cytoplasmic vacuoles, and scattered degenerated glial cells in fibrillary background with significant micro cyst development.

From FOS data, it is unclear which neuron populations participate in ejaculation and if neuronal activity causes or results in it. Thus, FOS cell density in immunohistochemistry increased in rats with induced fast ejaculation but dropped considerably after IN therapy in this research, indicating a strong association between FOS cell density and ejaculation speed. Dapoxetine inhibited ejaculation by reducing neuronal activity in the excitatory and hypothalamic areas. This research showed increased FOS cell density in the hippocampus and rat behaviour. Dapoxetine had no influence on FOS expression in sexually naïve male rats who had no interaction with females. Additionally, ejaculation-related brain regions showed little alterations following treatment. In our study, Fig. 9 and Table 6 show that dapoxetine per se contributed (21–30%) to the global diminution of FOS-positive cell density in rapid males in some structures and in others.

This research found that brain intranasal administration enhanced dapoxetine absorption. Nasal administration increased the bioavailability of liposomal dapoxetine. This study proves that intranasal CNS drug delivery is safe, efficacious, and simple. By reducing systemic exposure and boosting absorption, this approach may lessen medication side effects. It is reasonable to believe that nasal administration of a liposomal formulation may increase dapoxetine distribution in the central nervous system faster than other routes.

The comprehension of the effects of PE on the male and the sexual connection is contingent upon an understanding of the effects of PE on the companion, as multiple studies have indicated (Symonds et al. 2003; McMahon 2008; Metz and J.L.P., Michael 2000). If PE is a disease that affects both people and their relationships, partner needs must be addressed when judging severity and treatment results. Women reported pleasure with sexual engagement, interpersonal concerns, emotional anguish, and the man’s ejaculatory control.

A topical metered-dose aerosol anaesthetic spray with 2.5% lidocaine plus procaine or lidocaine alone. However, all genders have genital hypoesthesia (Busato and Galindo 2004). Metered-dose aerosol device with 2.5% lidocaine and procaine or lidocaine alone for topical use. Male and female genital hypoesthesia has been reported (Dinsmore and Wyllie 2009).

Current PE therapies include topical anaesthetics and oral medicines. In contrast to IN administration in this trial, oral medication includes SSRIs, PDE-5 inhibitors, antipsychotics, tricyclic antidepressants, and a1-adrenoreceptor antagonists. It takes a long time to work and has several side effects. Local anaesthetic therapy reduces mucous membrane sensitivity and irritation, but it alleviates symptoms. After determining the cause of acquired PE (e.g. hyperthyroidism), therapy might begin (Carani et al. 2005). Psychotherapy may be capable of alleviating the concurrent anxiety/depression and sexual issues (Carani et al. 2005). Topical anaesthetics may also cause penile hypo-anaesthesia and transvaginal absorption, which may cause vaginal numbness and female anorgasmia without a condom (Busato and Galindo 2004).

The DH-loaded PLGA nano formulation formed enhanced ejaculation delay, intromission frequency, mount frequency, latency, and penile erection index in this investigation. It also reduced post-ejaculatory delay. Additionally, FOS protein expression reduced, and genital smelling increased. This supports a previous study on DH and PE patients. First-dose dapoxetine of 30 or 60 mg 1.2 h before sexual activity was more effective than a placebo. Intra-ejaculation delay rose 2.5 and 3.0 times, ejaculatory control, distress reduction, and pleasure improved similarly (McMahon et al. 2011). So at our study, we tried to formulate DH to act rapidly through brain targeting through IN dosing also with higher efficacy and lower dose.

### Molecular docking simulation

However, docking investigations showed that the anticipated active sites of the tested receptors in brain receptors implicated in premature ejaculation had the greatest binding affinity or score, as indicated by a larger negative energy demand. CB-Dock2 was used to find interaction sites after obtaining proteins in pdb format. Additionally, common amino acids were considered. The grid box dimensions of each target protein were also established.

**Fig. 10:**
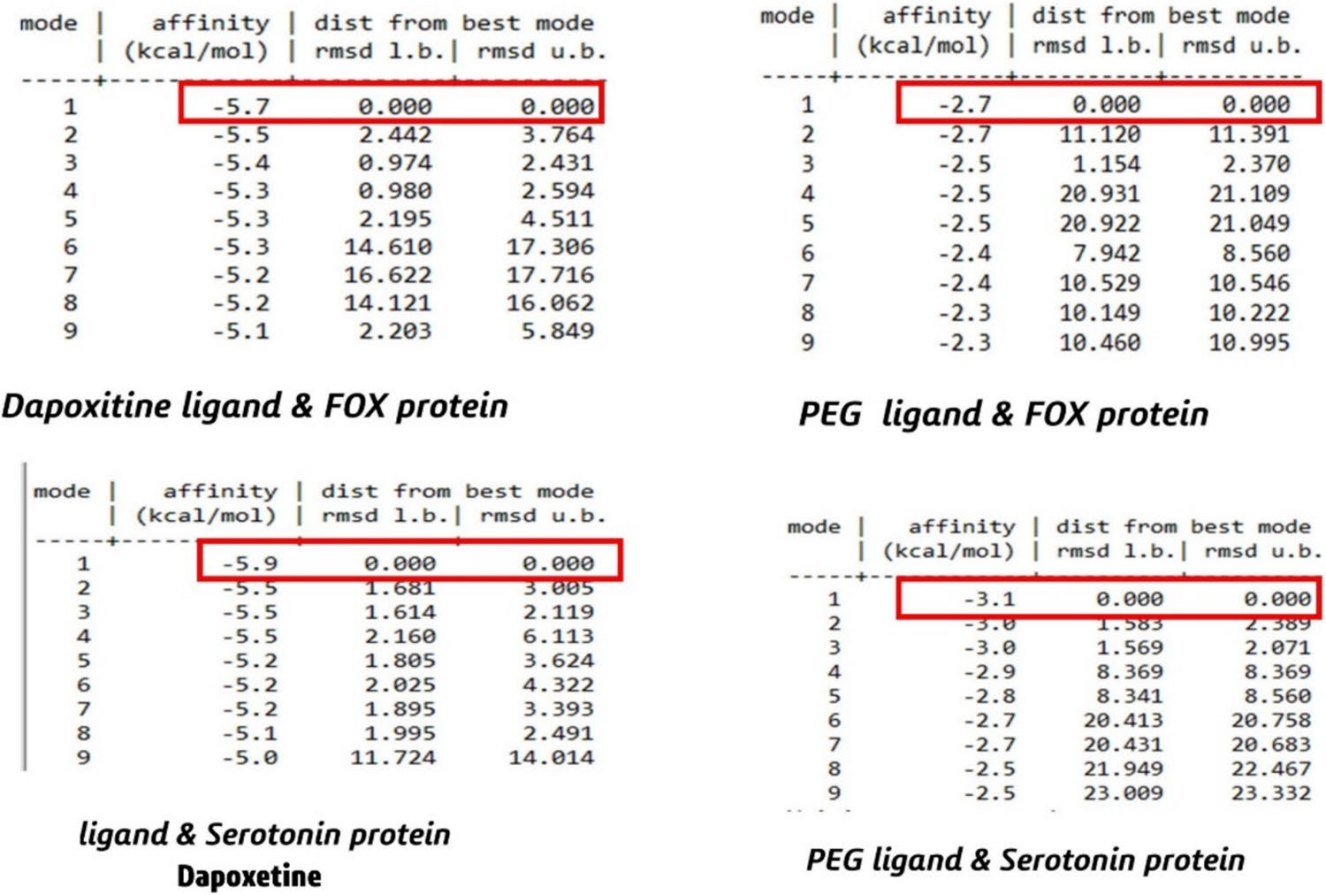
Binding affinity (kcal/mol) scoring degree of binding and interactions between DH and PLGA on serotonin and FOX receptors

DH and PLGA bound to Serotonin and FOX receptors with greater affinity (–5.9, –3.1, and –5.7, –2.7 kcal/mol). FOX was less active than serotonin for both ligands. They also had a higher affinity for PLGA (–2.9, –3.1) and (–1.5, –3.3) kcal/mol for the same receptor types. This caused regulated DH release following PLGA binding (Fig. 10). The active sites of a variety of targeted brain proteins were studied using molecular docking to determine the probable interaction mechanism between DH and PLGA and targeted proteins.

This study found that the targeted and chosen protein receptor and tested ligands form hydrogen, conventional, hydrophobic, and π-alkyl bonding interactions for DH and PLGA. Functional enrichment analysis identified many relationships between overrepresented protein domains in the network, since the method of determination determined the interaction categories. This suggests the system is using certain biological processes or pathways. Most complexes have hydrophobic and H-bonding interactions. Figures 11, 12, 13, 14, 15, 16, 17, and 18 are supplemented as Figs. S3–S10 show 3D and 2D docking sites between DH and serotonin. Most compounds exhibit H-bonding between the active side residues of the amide oxygen or nitrogen, albeit in a greater number of interactions, compared to DH and PLGA. DH and serotonin receptors bonded and interacted in S3. Fig. S4 shows DH-FOX 3D and 2D docking locations. Fig. S5 shows DH-FOX receptor bonding and interactions. Figures S6 and S7 depict 3D and 2D docking locations and bonding and interaction types between PLGA and serotonin receptors. Finally, Figs. S8 and S9 exhibited PLGA and FOX receptor 3D and 2D docking locations, while Fig. S10 illustrated their bonding and interaction kinds.

Serotonin and FOX receptor protein classes were tested for their principal identical interactions with DH and PLGA. This binding affinity and others to other proteins may be the main mechanism of DH and its PLGA coating for continuous release. The study found that DH has a high affinity for the active regions of several protein receptors.

The serotonin binding receptor’s active site bound affinities for DH and PLGA were − 5.9% and − 3.1 kcal/mol, whereas FOX receptors were − 5.7% and − 2.7. The ligand of active site 1 formed alkyl bonds with the receptor across an average distance of angstroms, according to further studies. Organic compounds have weak alkyl connections between homologous, nonreactive carbon groups. Pi-alkyl interactions between aromatic and aliphatic groups are characterized by the aromatic ring’s pi-electron density and the alkyl group’s electron density. These interactions show that strong hydrophobic bonds, non-covalent van der Waals interactions, and H. bonds contribute to the compound’s binding affinity. Atomic or molecular electron density changes start hydrogen bonding, a weak connection. The above interactions show that alpha guanine may form a stable complex with H. van der Waals forces and bonding.

In the mechanistic method, a synergist chemical is managed to enhance DH’s anticancer action and target brain receptors or proteins, which causes premature ejaculation. Our studies with the serotonin and FOX-functionalized four-arm PLGA showed that PLGA-coated DH increases DH activity, bioactivity, adhesive performance, and effectiveness. Common in humans, ejaculatory threshold abnormalities, including persistent premature ejaculation, may lower quality of life. Human and animal studies reveal that greater central nervous system serotonin levels raise the ejaculatory threshold, whereas depletion decreases it (Jong et al. 2006). We examined DH’s binding affinity with serotonin receptors to demonstrate that serotonin levels are likely reduced. Like the FOX protein, this protein type encodes a transcription factor that regulates many target genes, including those involved in sex development, eyelid development, ovarian function and maintenance, genomic integrity, and cellular pathways like cell-cycle progression, proliferation, and apoptosis (Tucker 2022). So, at this study both DH and PLGA showed affinity towards the FOX protein.

The study evaluates the binding effectiveness of DH and PLGA as experimentally bound to receptors by calculating binding affinity and free energy (ΔG). The binding site and interaction were thoroughly depicted, and all aspects that increase these interactions and binding effectiveness were searched for. Understanding the elements that enhance these interactions and their binding locations and interactions helps identify potentially successful medicines from the large pool of natural product and repurposing drug candidates. Computational chemistry, molecular docking, and in silico toxicity may help determine ligand-receptor binding reactivity. Additionally, the discovered binding locations and methods of action are explored.

Next, this work used molecular docking to accurately define the mechanism of action and separate route for DH activity. This research examined DH’s in vitro effectiveness against serotonin and FOX receptors and its link with the suppression of key proteins that cause premature ejaculation in normal and diabetic rats.

Our findings imply that DH and PLGA contribute to premature ejaculation in normal and diabetic animals. Many proteins will be affected. However, further study is needed to understand the molecular processes of DH action and evaluate its potential as a premature ejaculation issue treatment. Recently, the X-ray diffraction crystalline specific functions receptor. The inhibitory affinity (pKd and ΔG) of DH and PLGA were evaluated. Results showed DH and PLGA had a much higher receptor affinity. Analysis identified the active site’s binding-critical amino acid residues (as decided). Interactions include hydrophobic and hydrogen bonding.

## Conclusions and recommendations

Passive focused intranasal treatment treats men’s rapid ejaculations with long-term benefits in this study. The safe, well-tolerated IN drug combination delays quick ejaculation by targeting the brain. The greatest dapoxetine effectiveness and safety database for PE men, especially after Nano therapy preparation, is available. Intranasal dapoxetine dispersion was constant, and high-EE and sustained-release liposomes increased brain bioavailability. In rats, acute dapoxetine prevented quick ejaculation and changed contextual neuronal activity in ejaculatory network brain areas. Acute dapoxetine slows PE rat ejaculation. These results enhance PE neurobiology and pharmacology investigations. Targeting many medications to the brain may have potential therapeutic applications. This method will help cure Alzheimer’s, brain tumors, Parkinson’s, schizophrenia, and sleep difficulties. In future, the present study needs to be validated through estimation of blood and brain targeting efficiency pharmacokinetics in a suitable animal model. Our study shows that DH and PLGA contribute to premature ejaculation in normal and diabetic animals. This will affect several proteins (serotonin and FOX receptors).

### Limitations and future respective

Pharmacokinetic analysis study will be performed in both serum and brain for detailed kinetic parameters and bioavailability percentages reach to the brain in future; respective will be estimated in addition to the safety systematic evaluation.

## Supplementary Information

Below is the link to the electronic supplementary material.

ESM1 (ZIP 8.38 MB)

## Data Availability

All source data for this work (or generated in this study) are available upon reasonable request.
