# Supplementary figures and images for: Nose to brain delivery of dapoxetine-loaded PLGA nanoparticle for treatment of premature ejaculation distress in normal and diabetic rats with in silico targeting brain FOX and serotonin proteins
Source: Naunyn Schmiedebergs Arch Pharmacol. 2025 Dec 23;399(6):8131–54. doi: 10.1007/s00210-025-04876-4 (PMC13086728; doi:10.1007/s00210-025-04876-4)

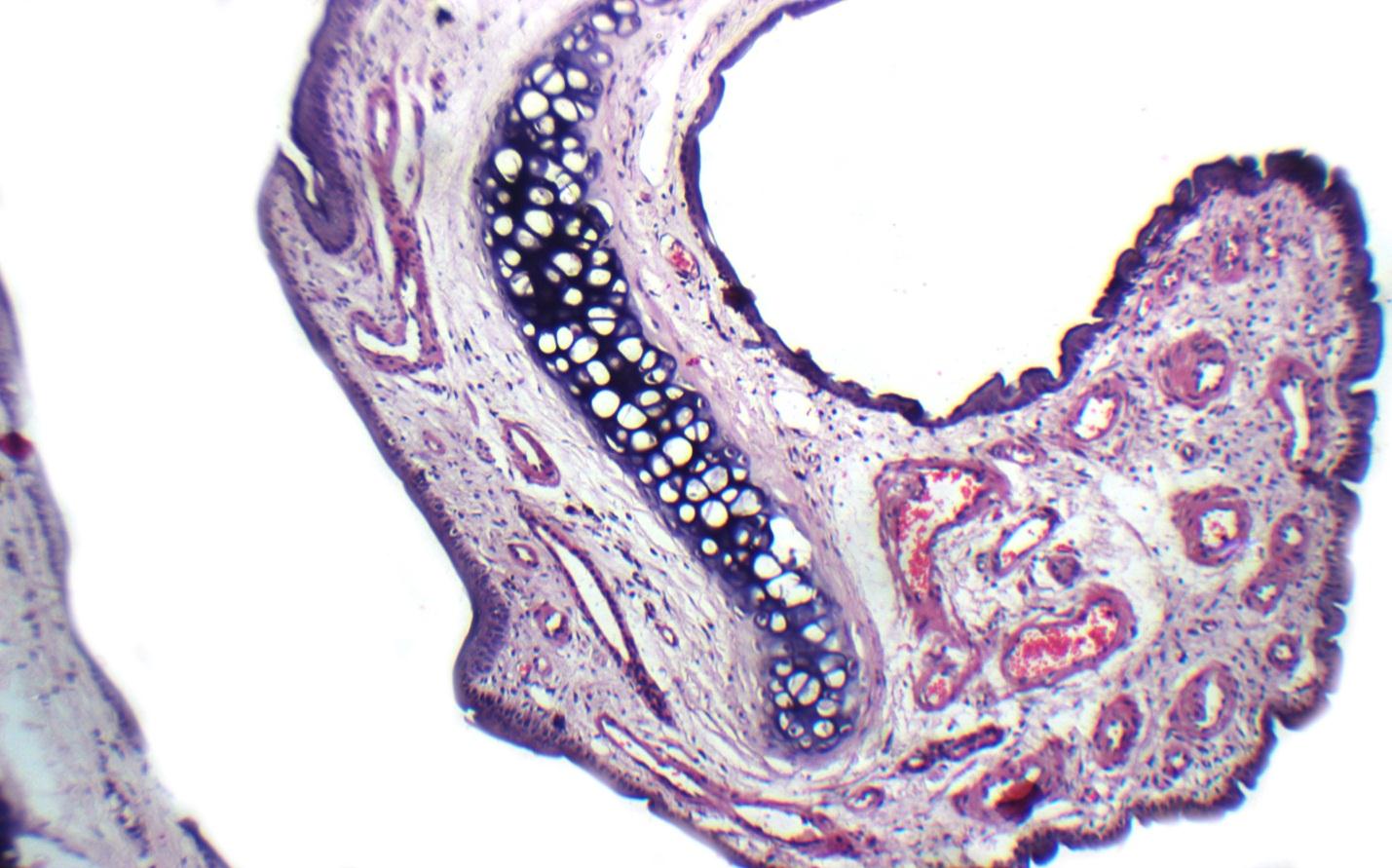

Supplement: Supplementary file 1 — (ZIP 8.38 MB) [file 210_2025_4876_MOESM1_ESM.zip › control 1.jpg]

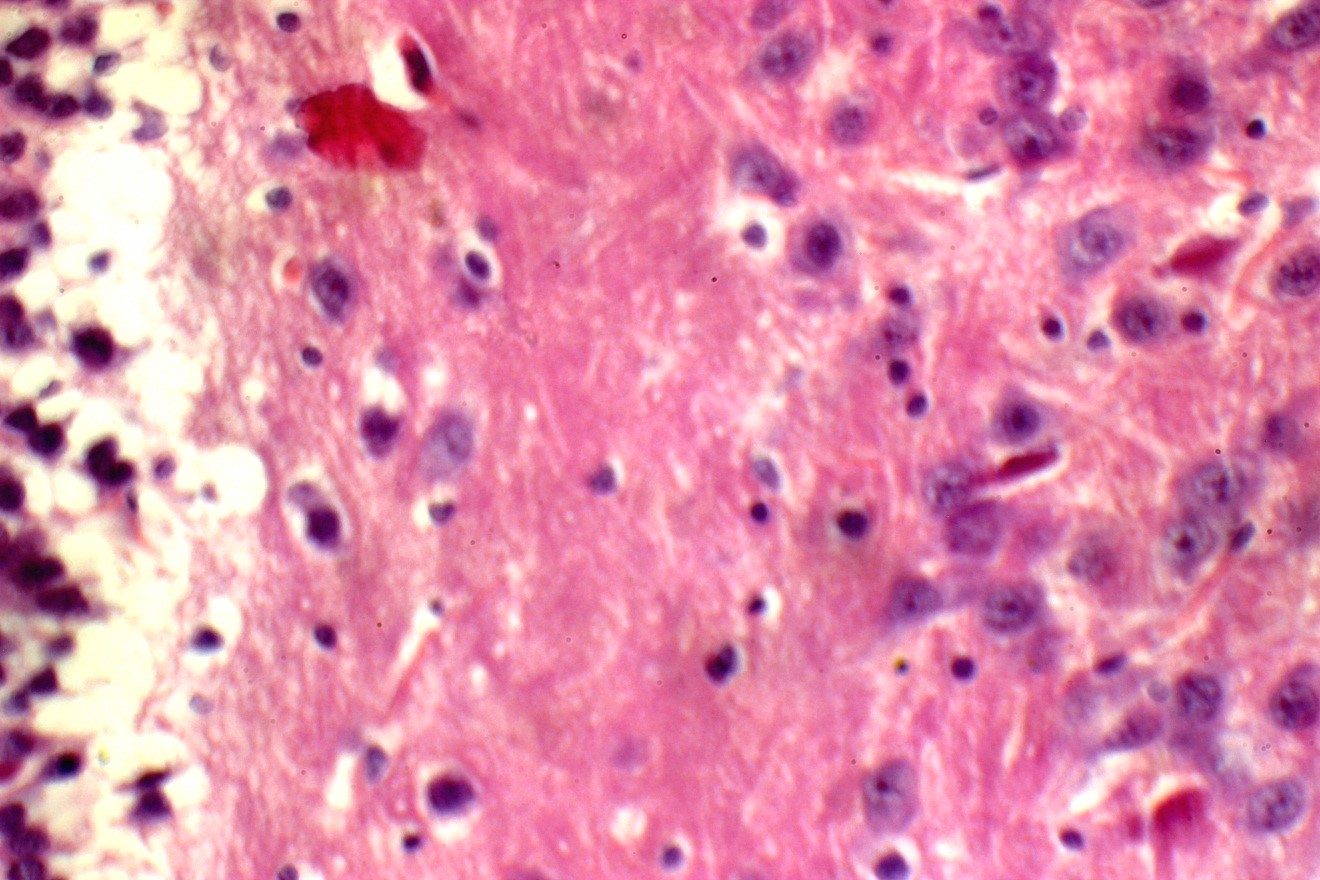

Supplement: Supplementary file 1 — (ZIP 8.38 MB) [file 210_2025_4876_MOESM1_ESM.zip › D..... Hippo.jpg]

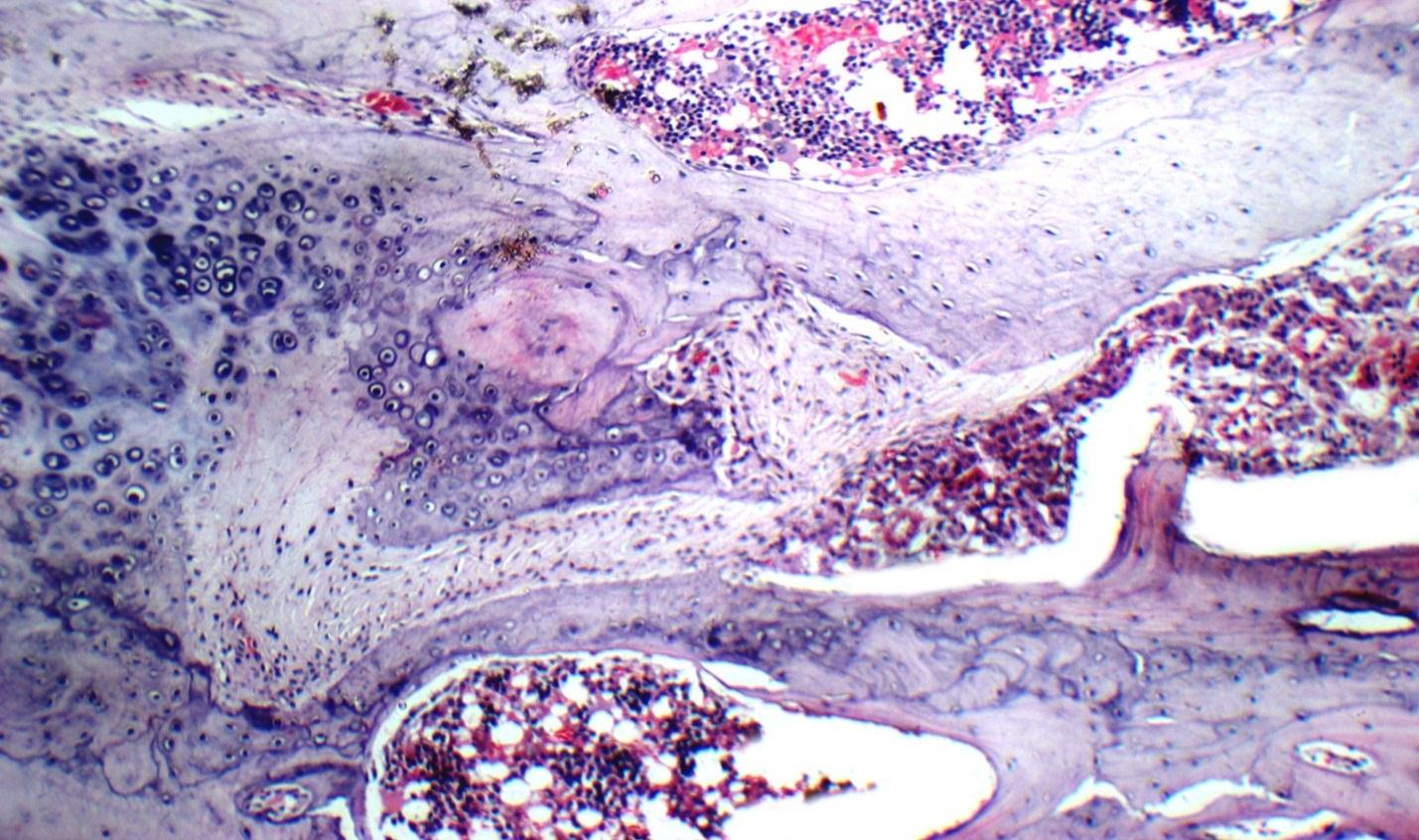

Supplement: Supplementary file 1 — (ZIP 8.38 MB) [file 210_2025_4876_MOESM1_ESM.zip › diabetic, IN.jpg]

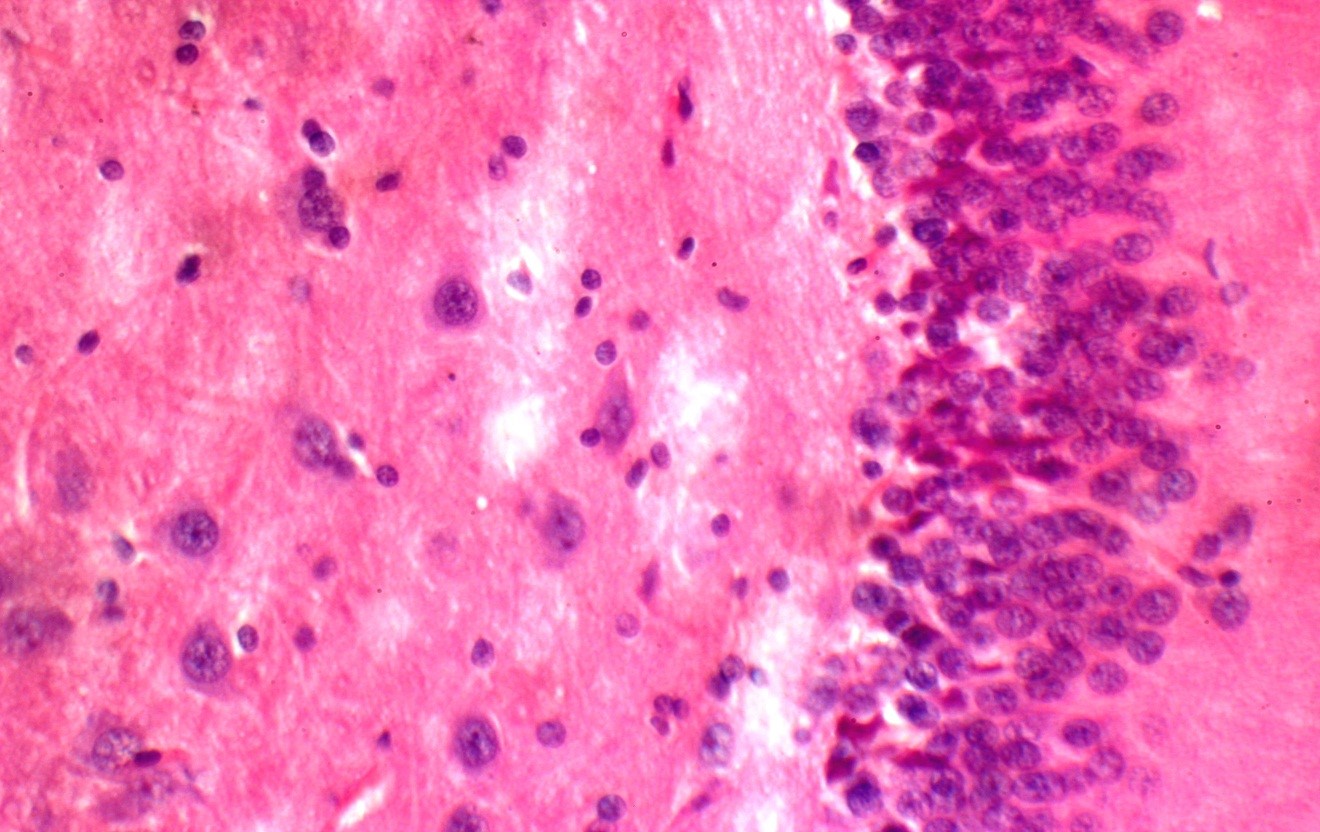

Supplement: Supplementary file 1 — (ZIP 8.38 MB) [file 210_2025_4876_MOESM1_ESM.zip › E.... Hippo.jpg]

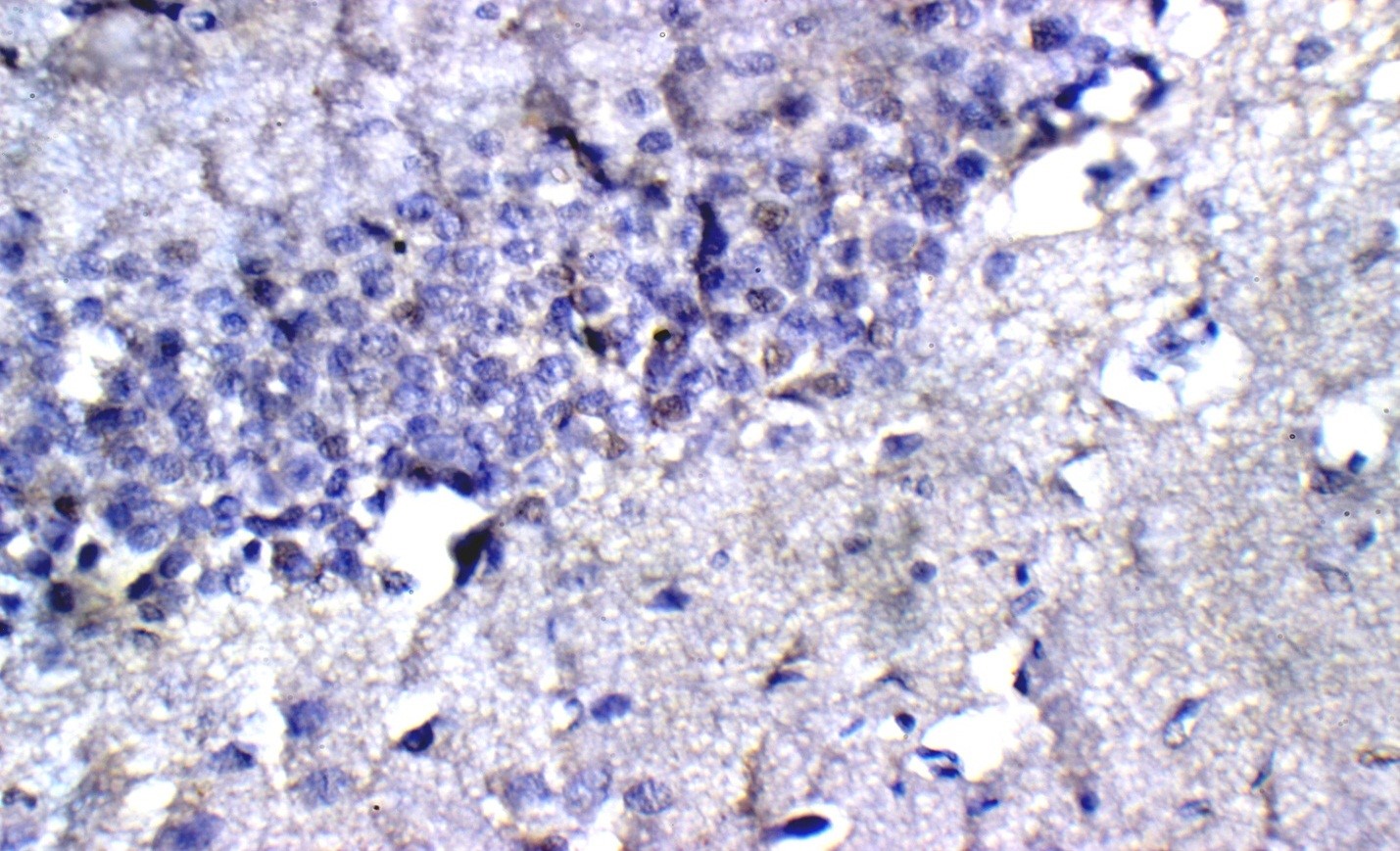

Supplement: Supplementary file 1 — (ZIP 8.38 MB) [file 210_2025_4876_MOESM1_ESM.zip › E.jpg]

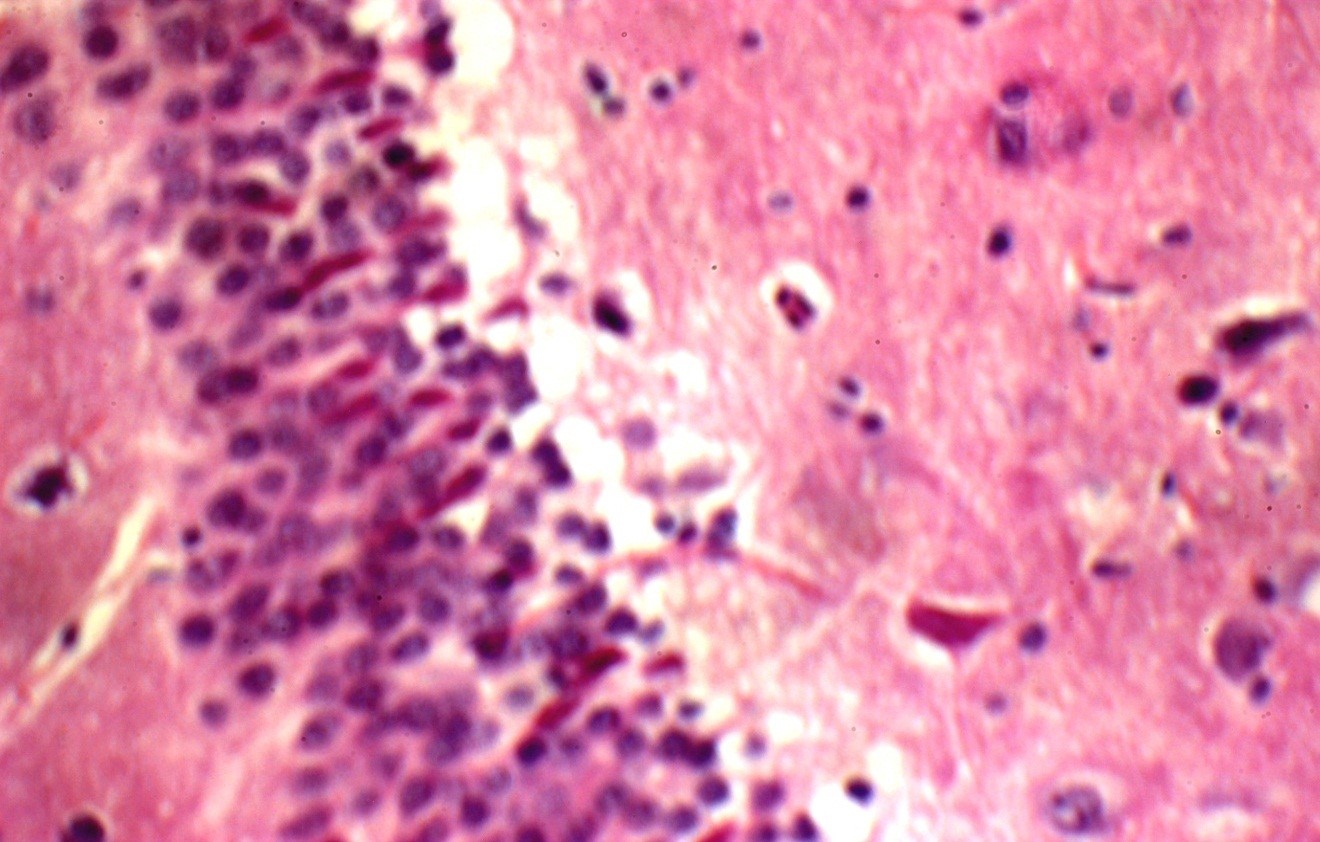

Supplement: Supplementary file 1 — (ZIP 8.38 MB) [file 210_2025_4876_MOESM1_ESM.zip › F..... Hippo.jpg]

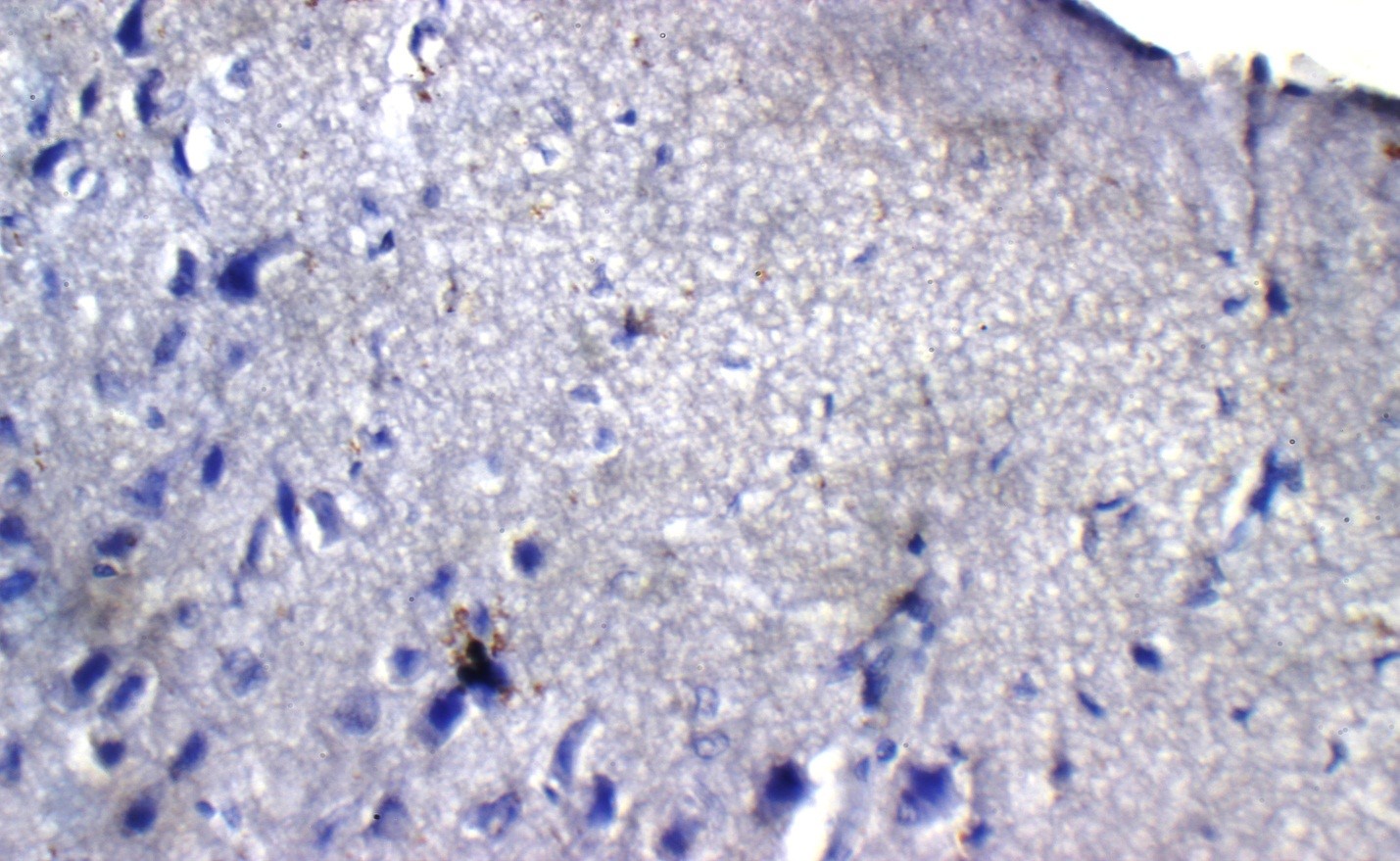

Supplement: Supplementary file 1 — (ZIP 8.38 MB) [file 210_2025_4876_MOESM1_ESM.zip › F.jpg]

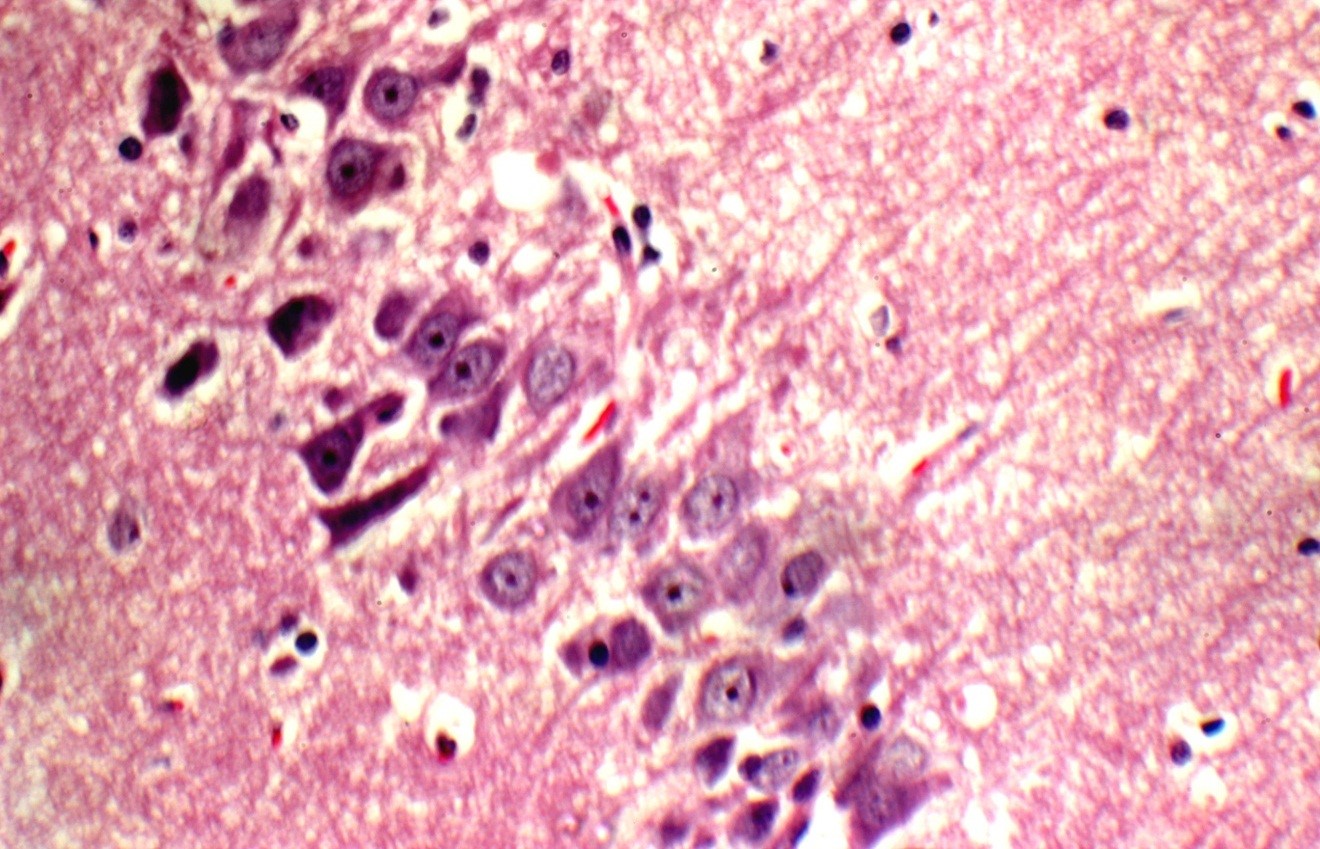

Supplement: Supplementary file 1 — (ZIP 8.38 MB) [file 210_2025_4876_MOESM1_ESM.zip › G..... Hippo.jpg]

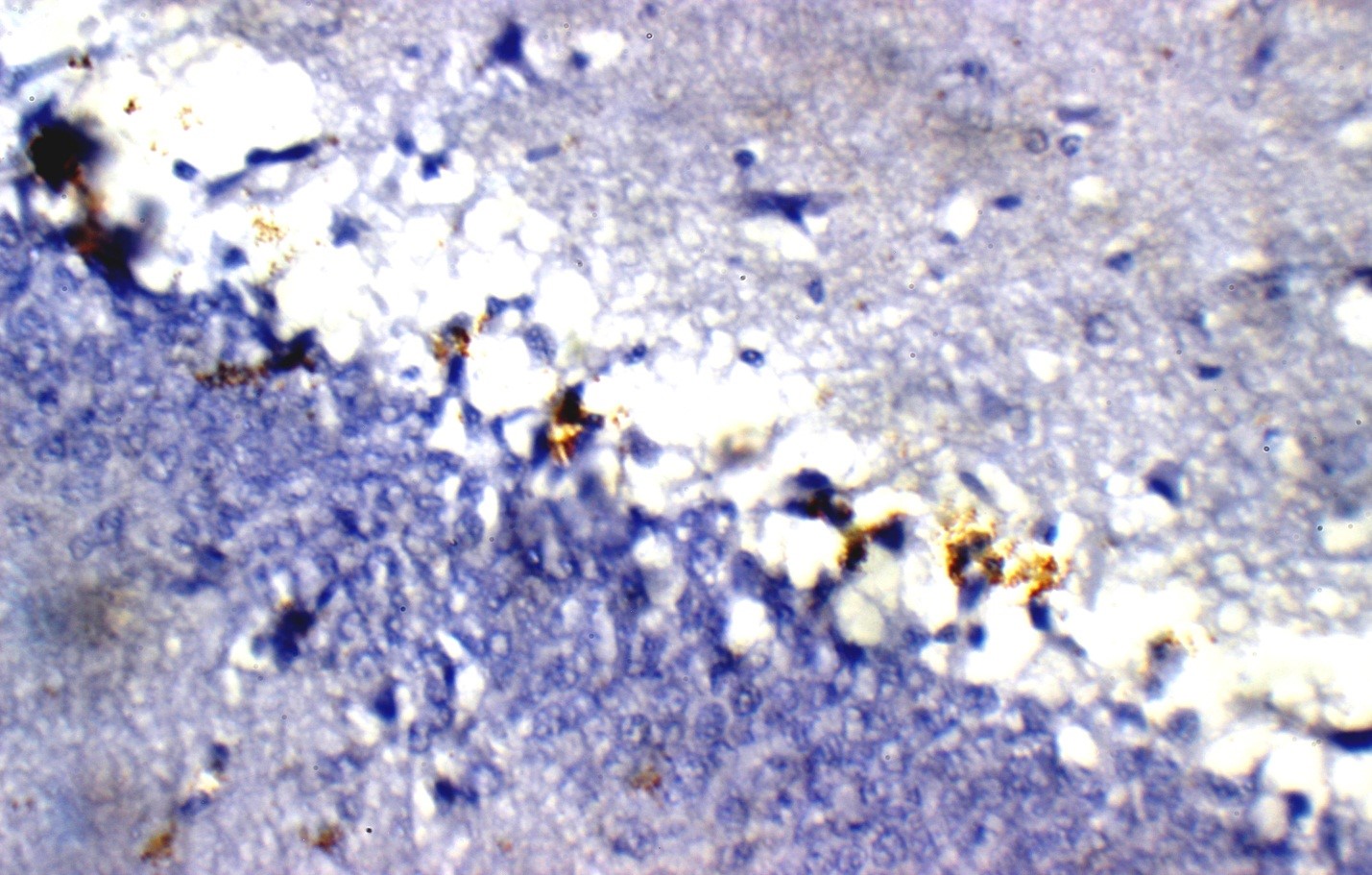

Supplement: Supplementary file 1 — (ZIP 8.38 MB) [file 210_2025_4876_MOESM1_ESM.zip › G.jpg]

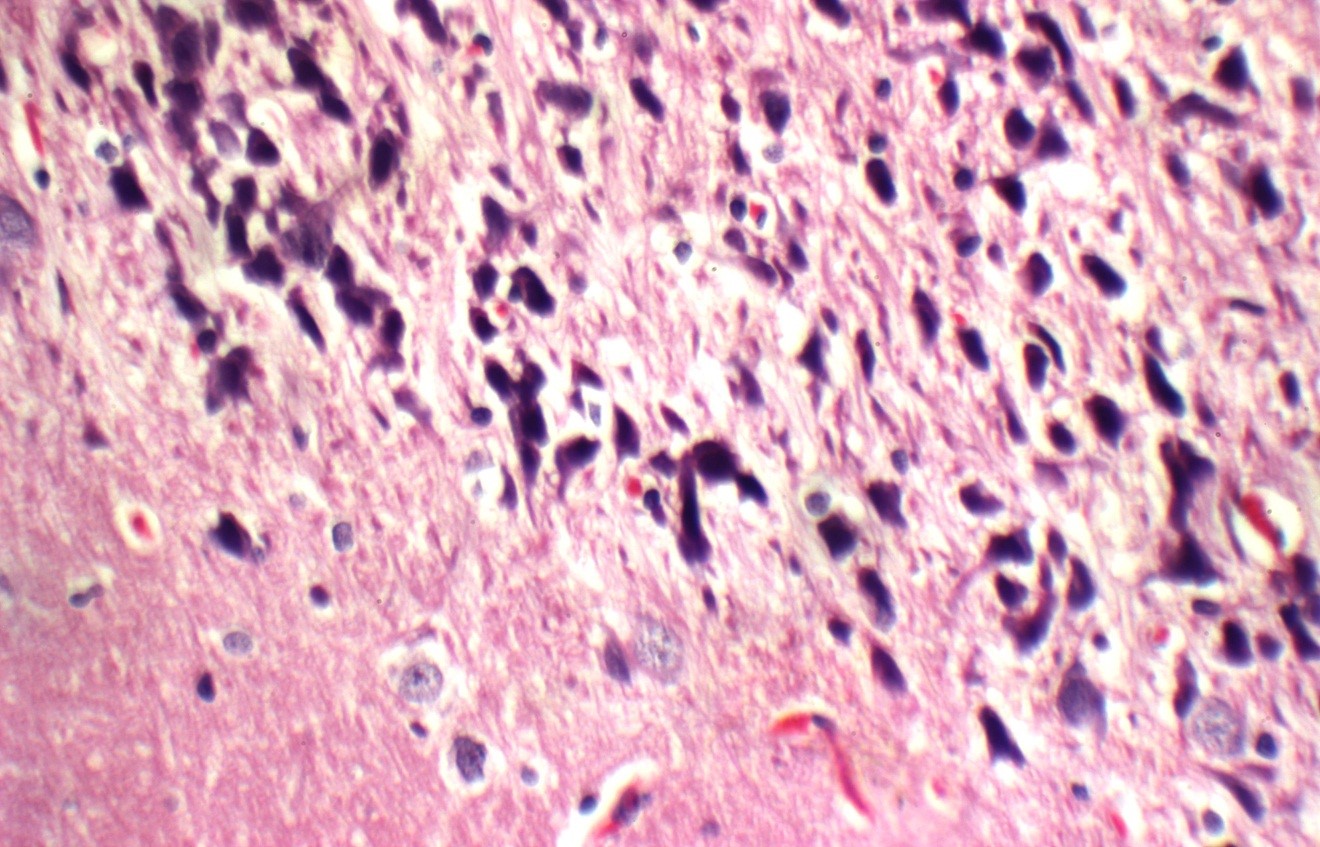

Supplement: Supplementary file 1 — (ZIP 8.38 MB) [file 210_2025_4876_MOESM1_ESM.zip › H..... Hippo.jpg]

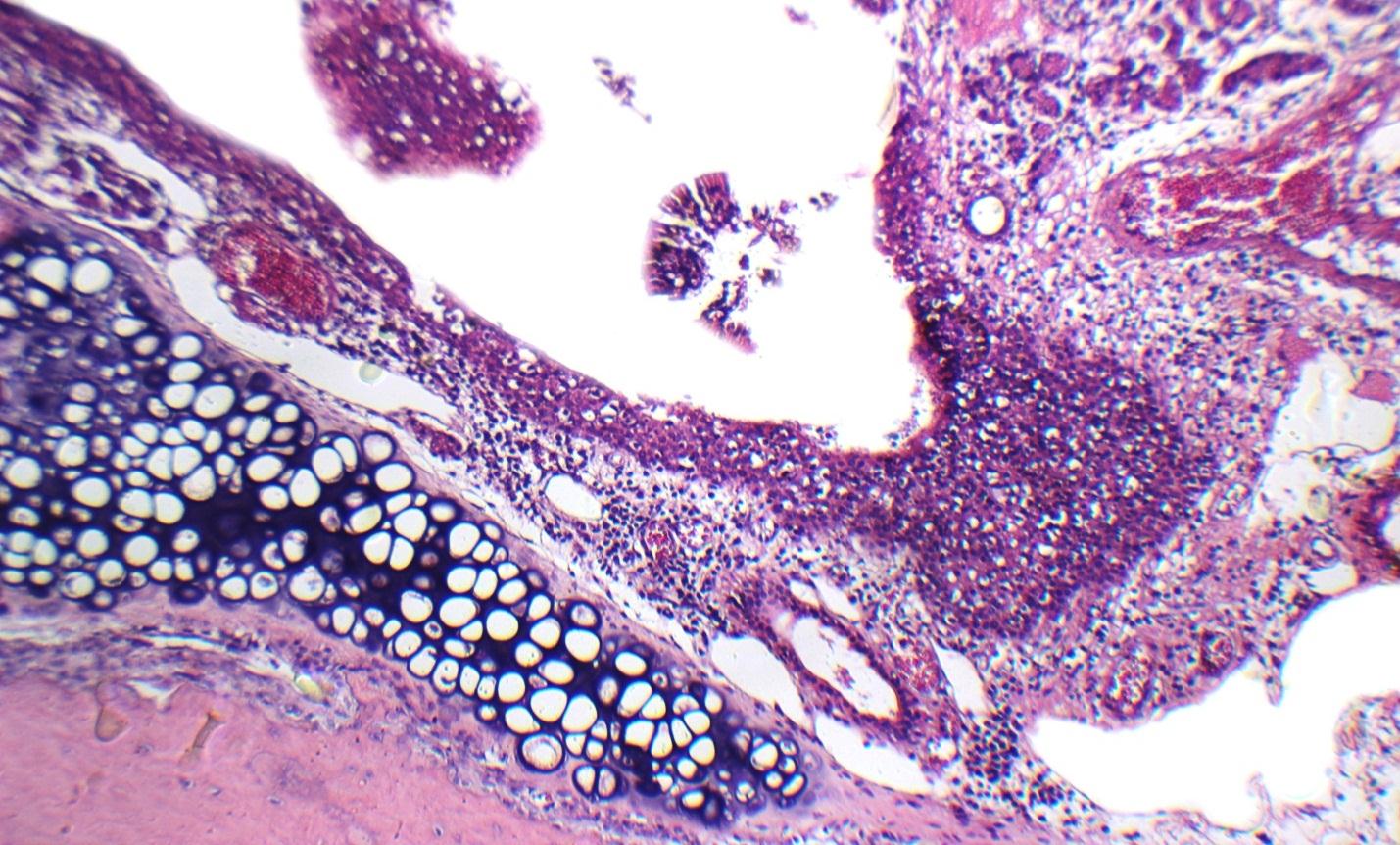

Supplement: Supplementary file 1 — (ZIP 8.38 MB) [file 210_2025_4876_MOESM1_ESM.zip › PE, I-N.jpg]

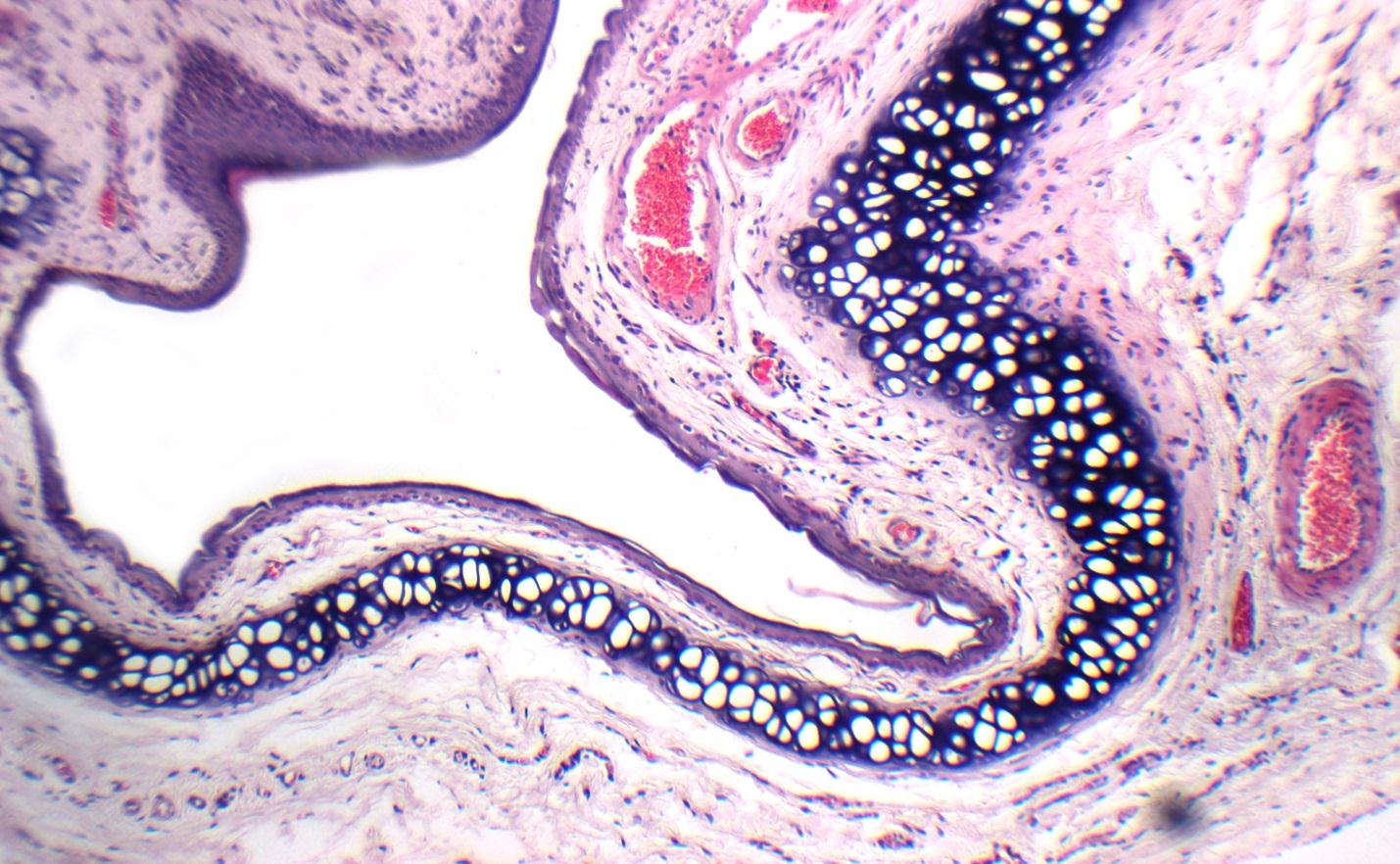

Supplement: Supplementary file 1 — (ZIP 8.38 MB) [file 210_2025_4876_MOESM1_ESM.zip › stndard 2.jpg]

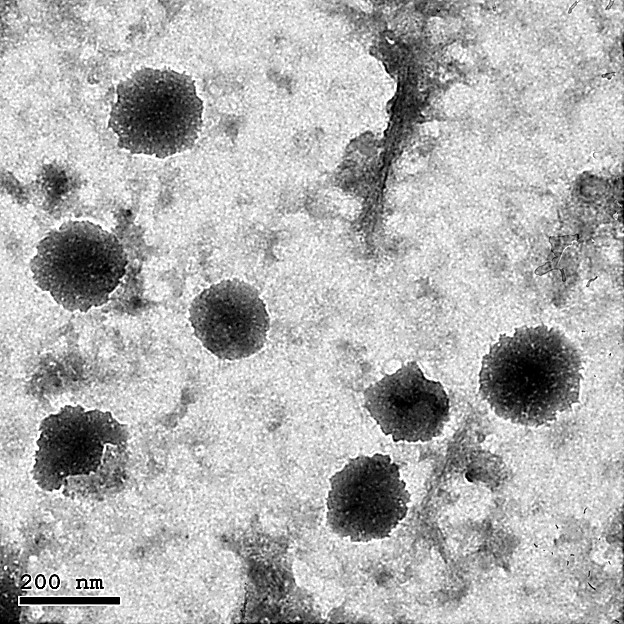

Supplement: Supplementary file 1 — (ZIP 8.38 MB) [file 210_2025_4876_MOESM1_ESM.zip › TEM.jpg]

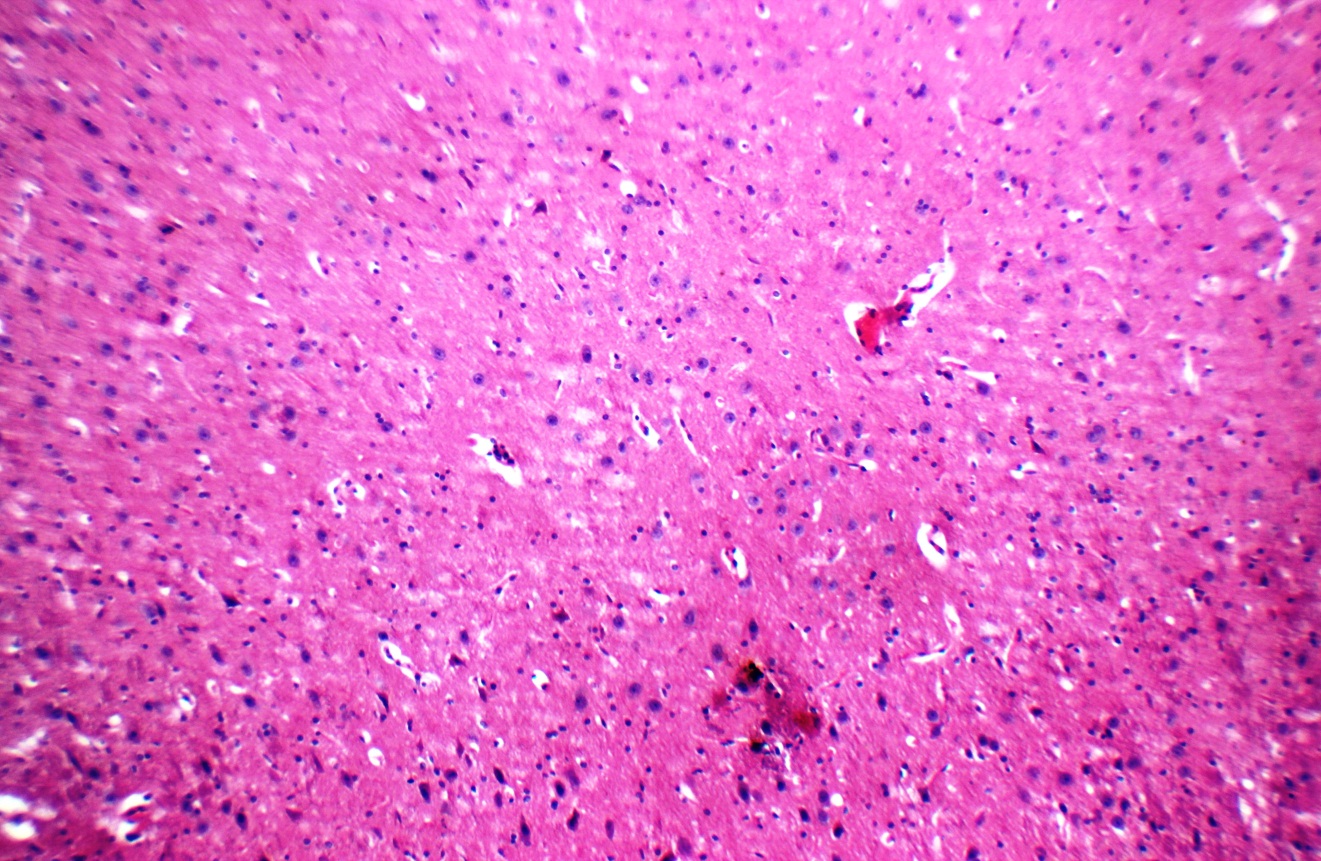

Supplement: Supplementary file 1 — (ZIP 8.38 MB) [file 210_2025_4876_MOESM1_ESM.zip › 1.A, Control.jpg]

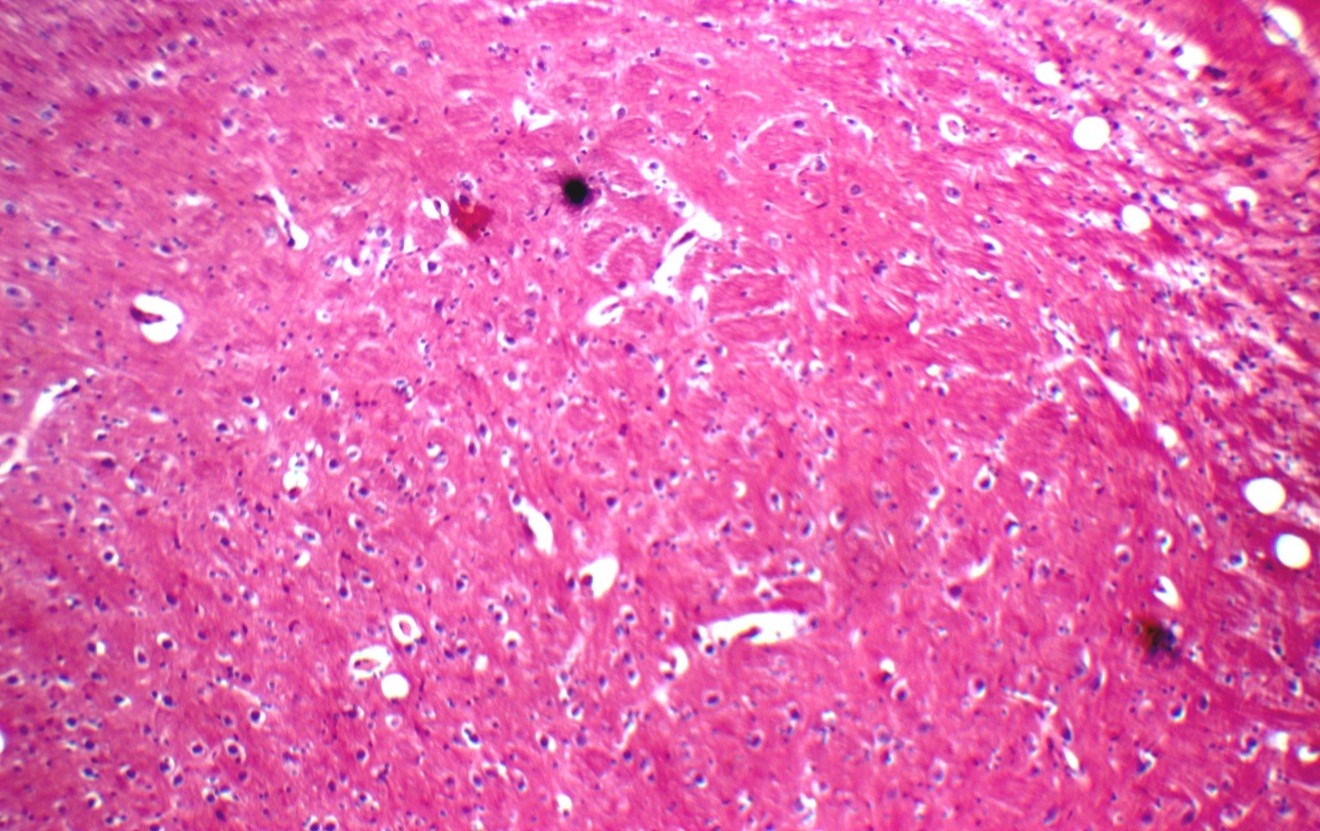

Supplement: Supplementary file 1 — (ZIP 8.38 MB) [file 210_2025_4876_MOESM1_ESM.zip › 2. B... diabetic.jpg]

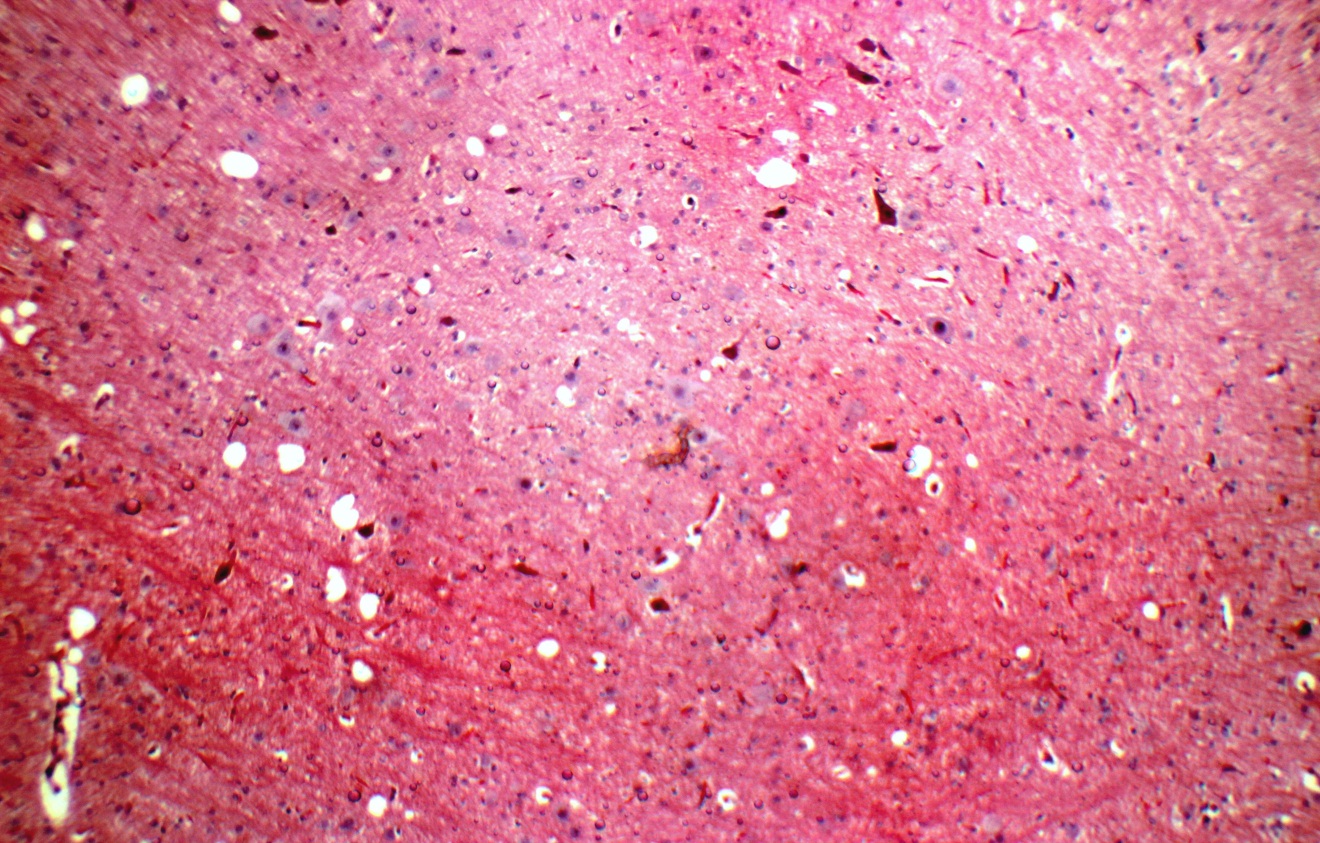

Supplement: Supplementary file 1 — (ZIP 8.38 MB) [file 210_2025_4876_MOESM1_ESM.zip › 3. C..... PE.jpg]

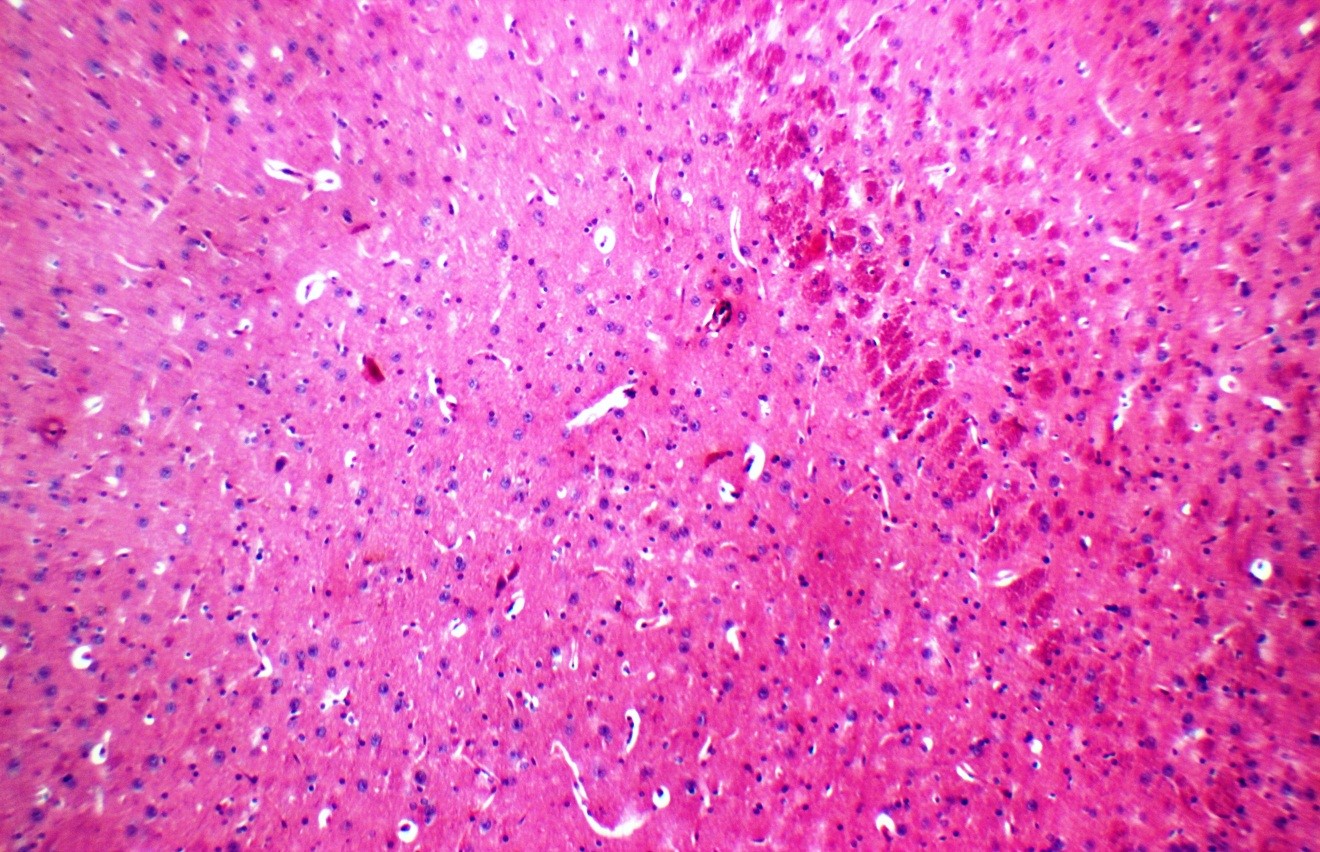

Supplement: Supplementary file 1 — (ZIP 8.38 MB) [file 210_2025_4876_MOESM1_ESM.zip › 4. D.... brain.jpg]

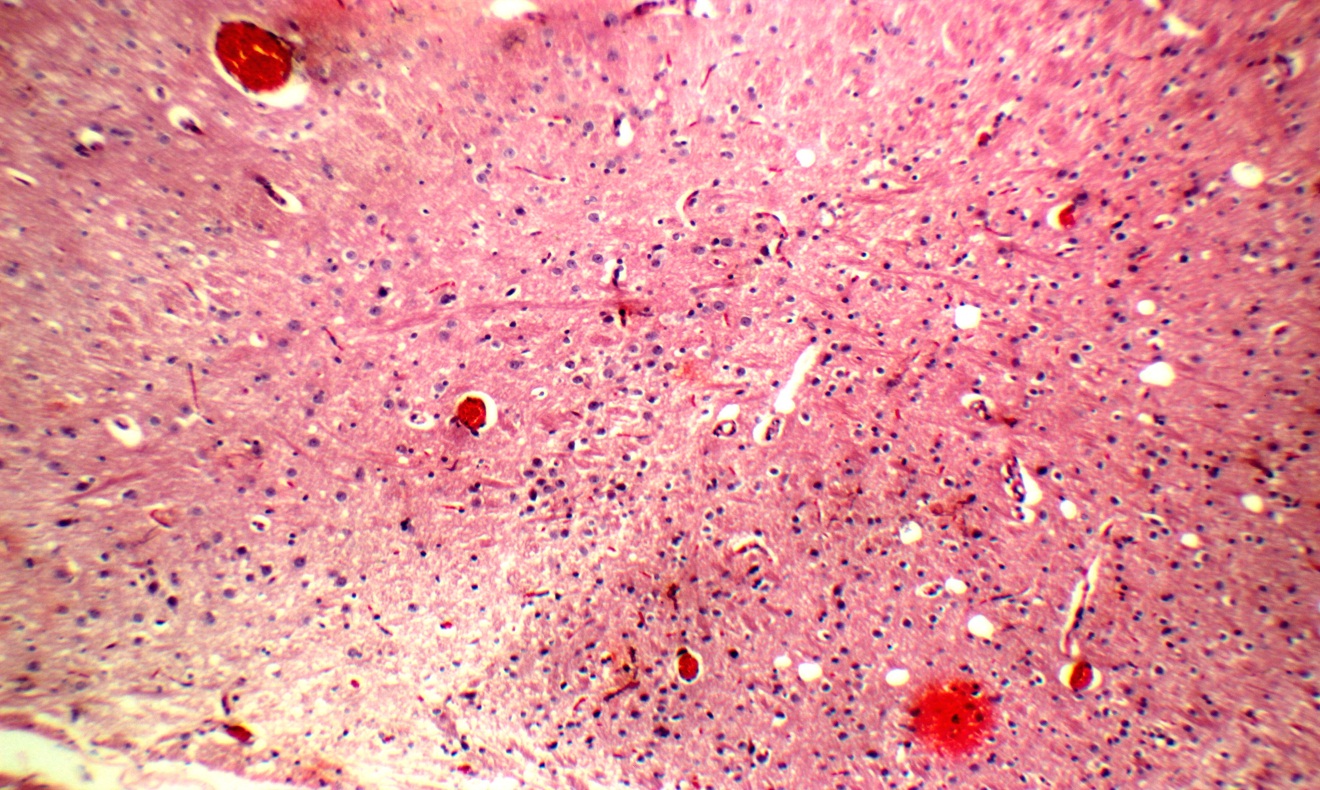

Supplement: Supplementary file 1 — (ZIP 8.38 MB) [file 210_2025_4876_MOESM1_ESM.zip › 5. E.....diab, ttt oral, PE.jpg]

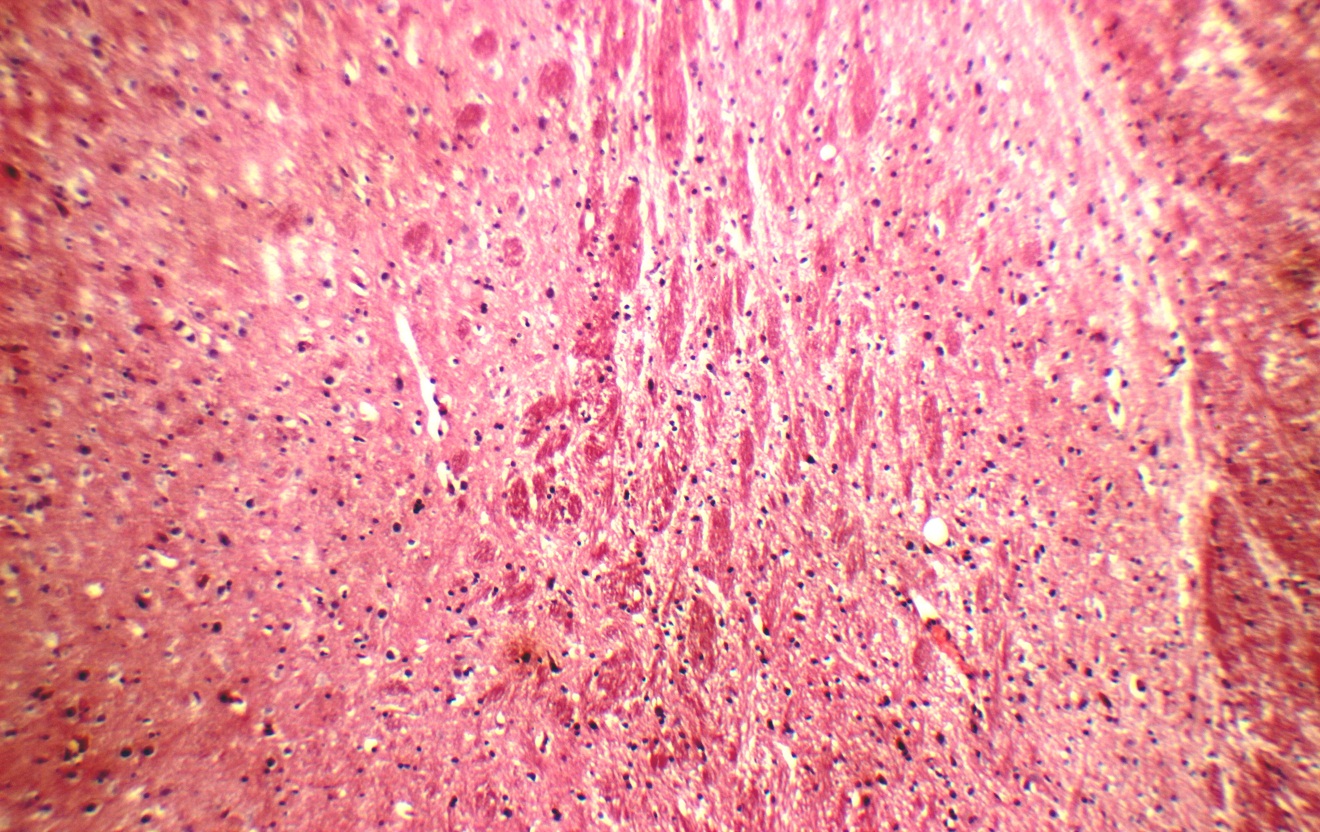

Supplement: Supplementary file 1 — (ZIP 8.38 MB) [file 210_2025_4876_MOESM1_ESM.zip › 6. F..... diabetic, ttt IN.jpg]

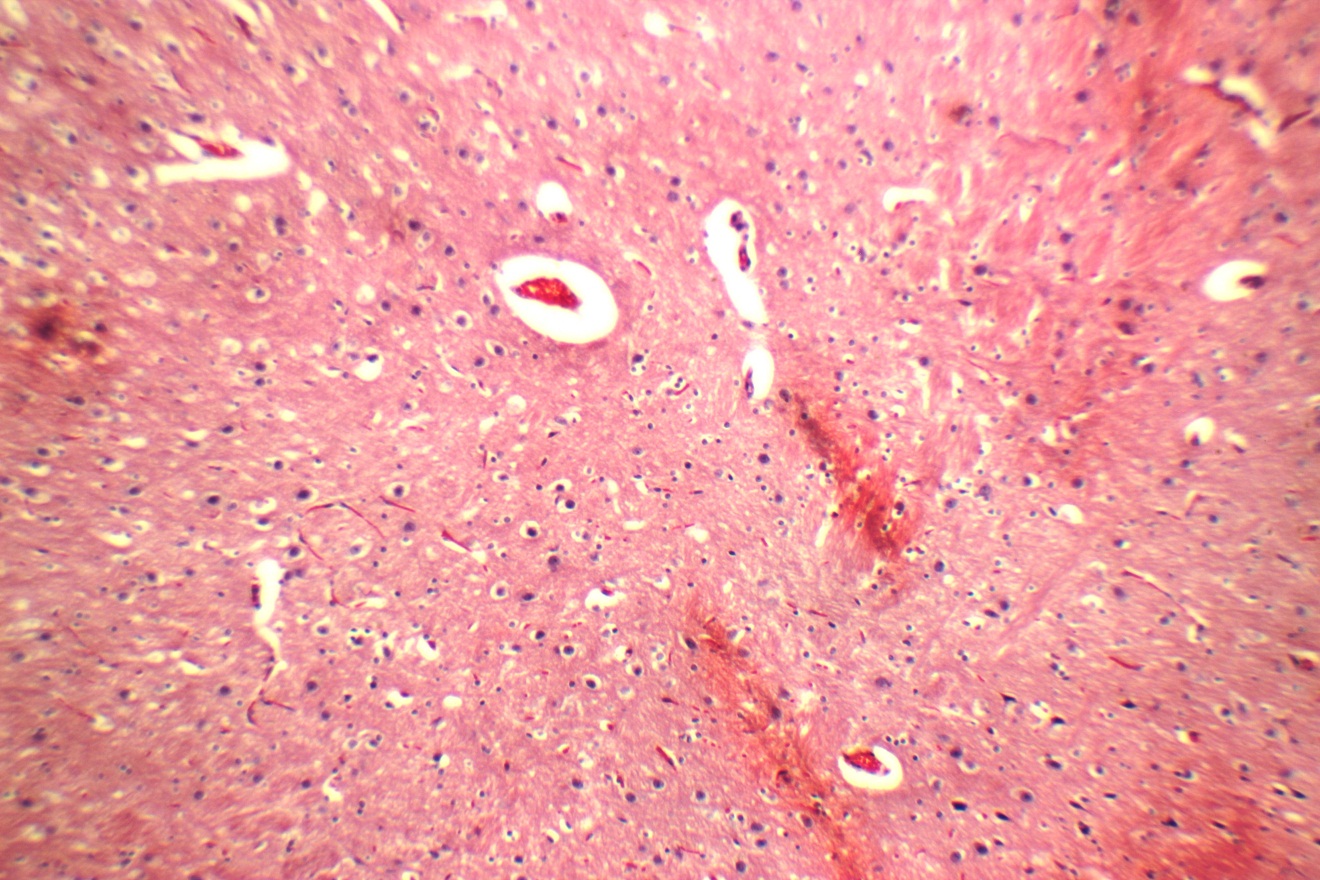

Supplement: Supplementary file 1 — (ZIP 8.38 MB) [file 210_2025_4876_MOESM1_ESM.zip › 7.G.... PE. IN,.jpg]

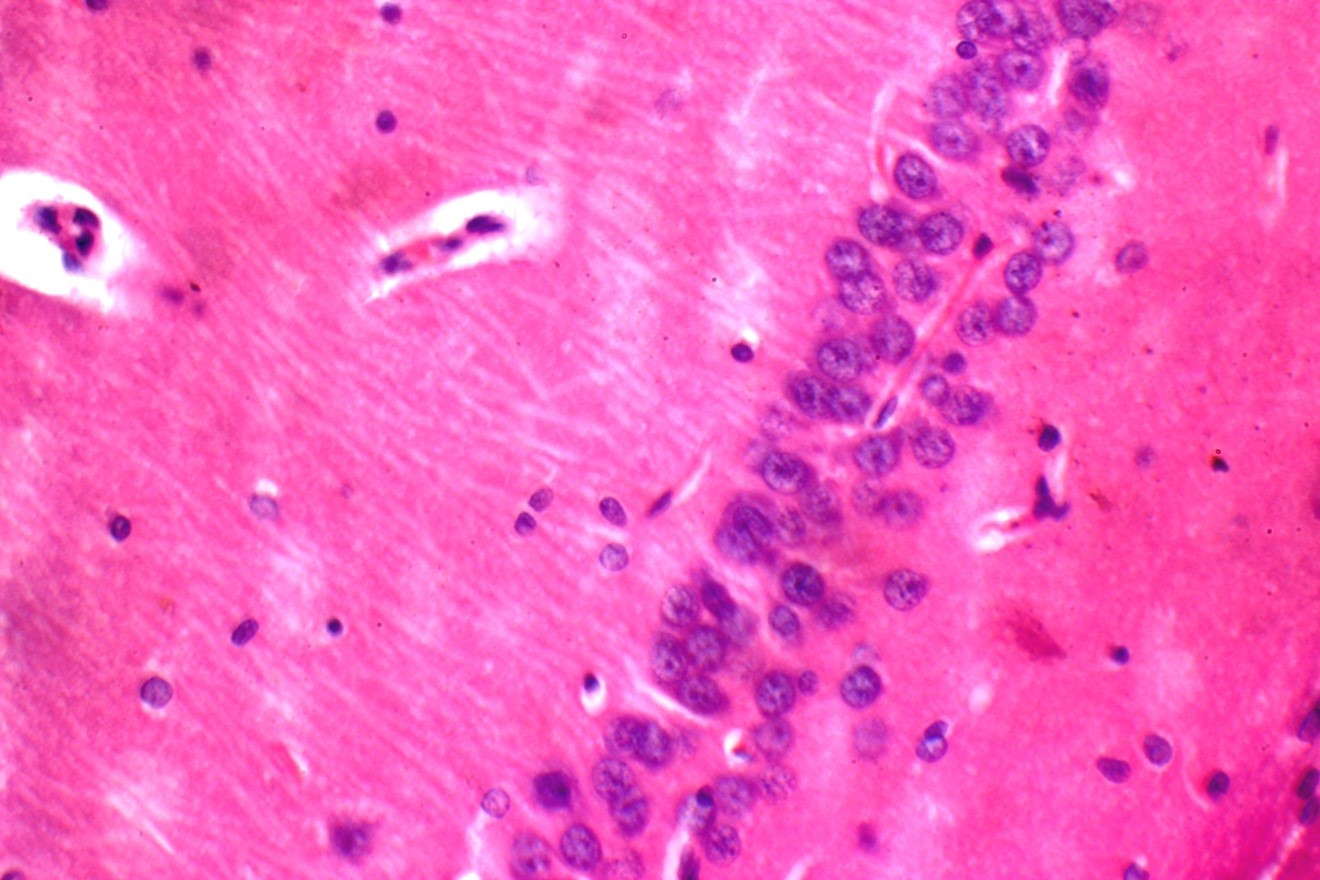

Supplement: Supplementary file 1 — (ZIP 8.38 MB) [file 210_2025_4876_MOESM1_ESM.zip › A.... Hippo.jpg]

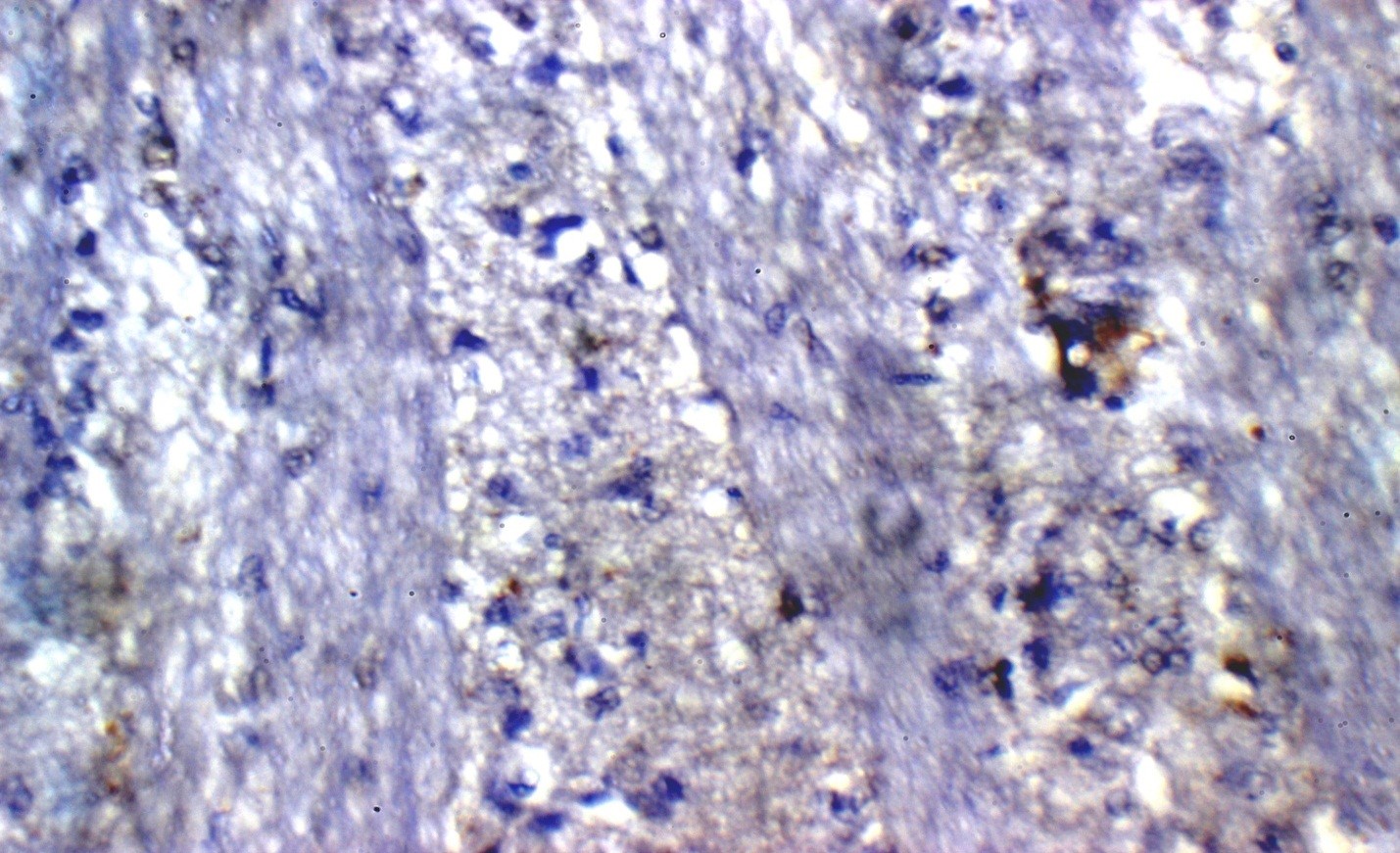

Supplement: Supplementary file 1 — (ZIP 8.38 MB) [file 210_2025_4876_MOESM1_ESM.zip › A.jpg]

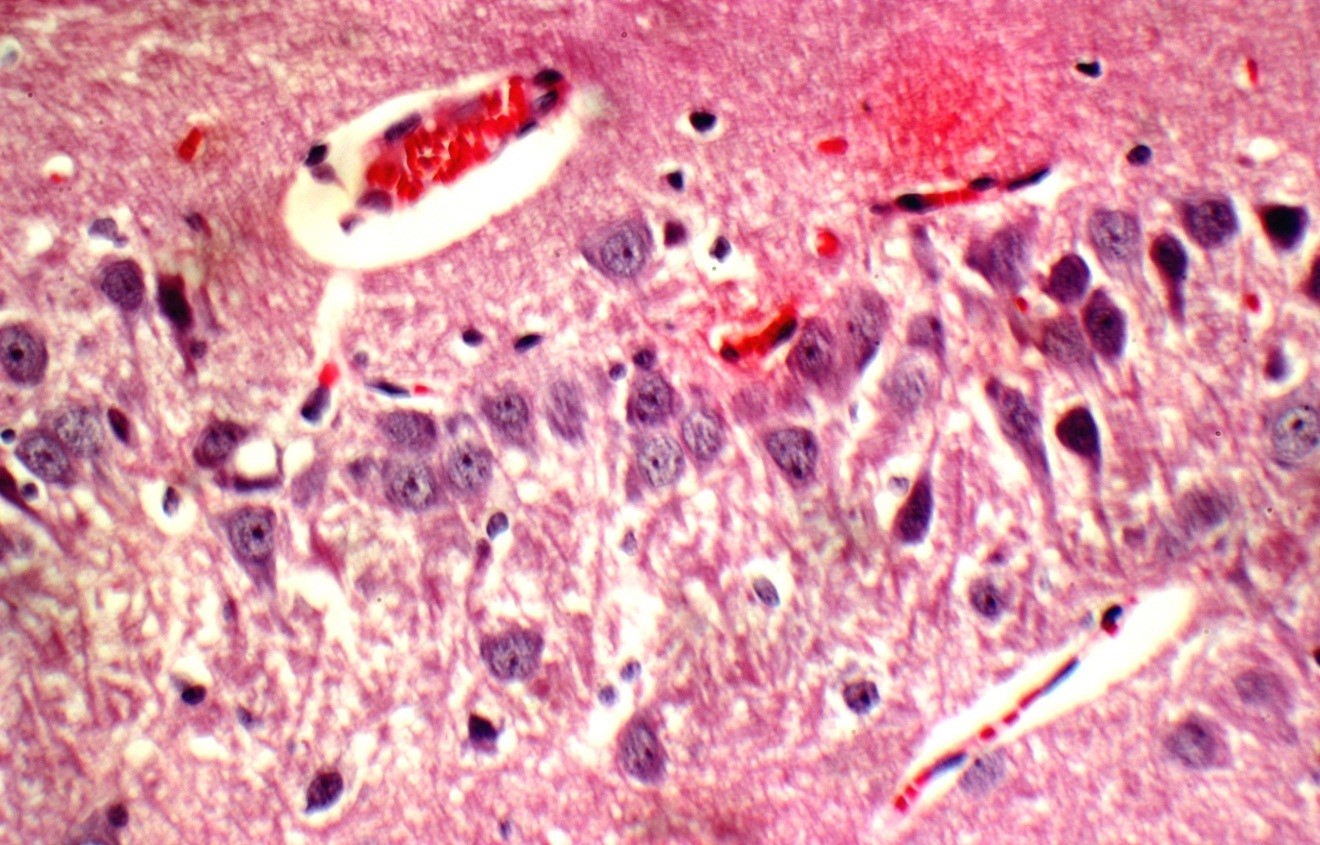

Supplement: Supplementary file 1 — (ZIP 8.38 MB) [file 210_2025_4876_MOESM1_ESM.zip › B origin Hippo.jpg]

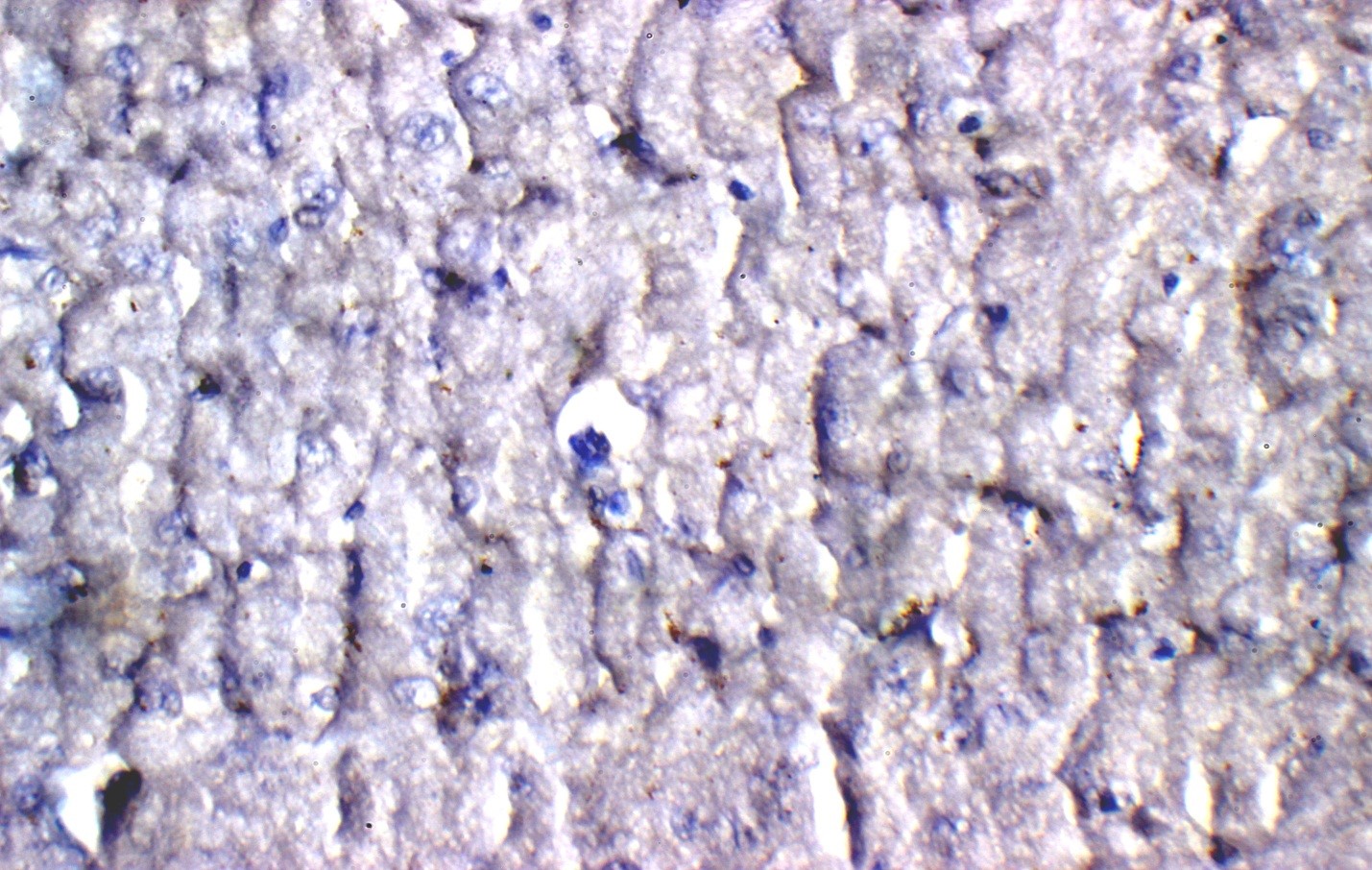

Supplement: Supplementary file 1 — (ZIP 8.38 MB) [file 210_2025_4876_MOESM1_ESM.zip › B.jpg]

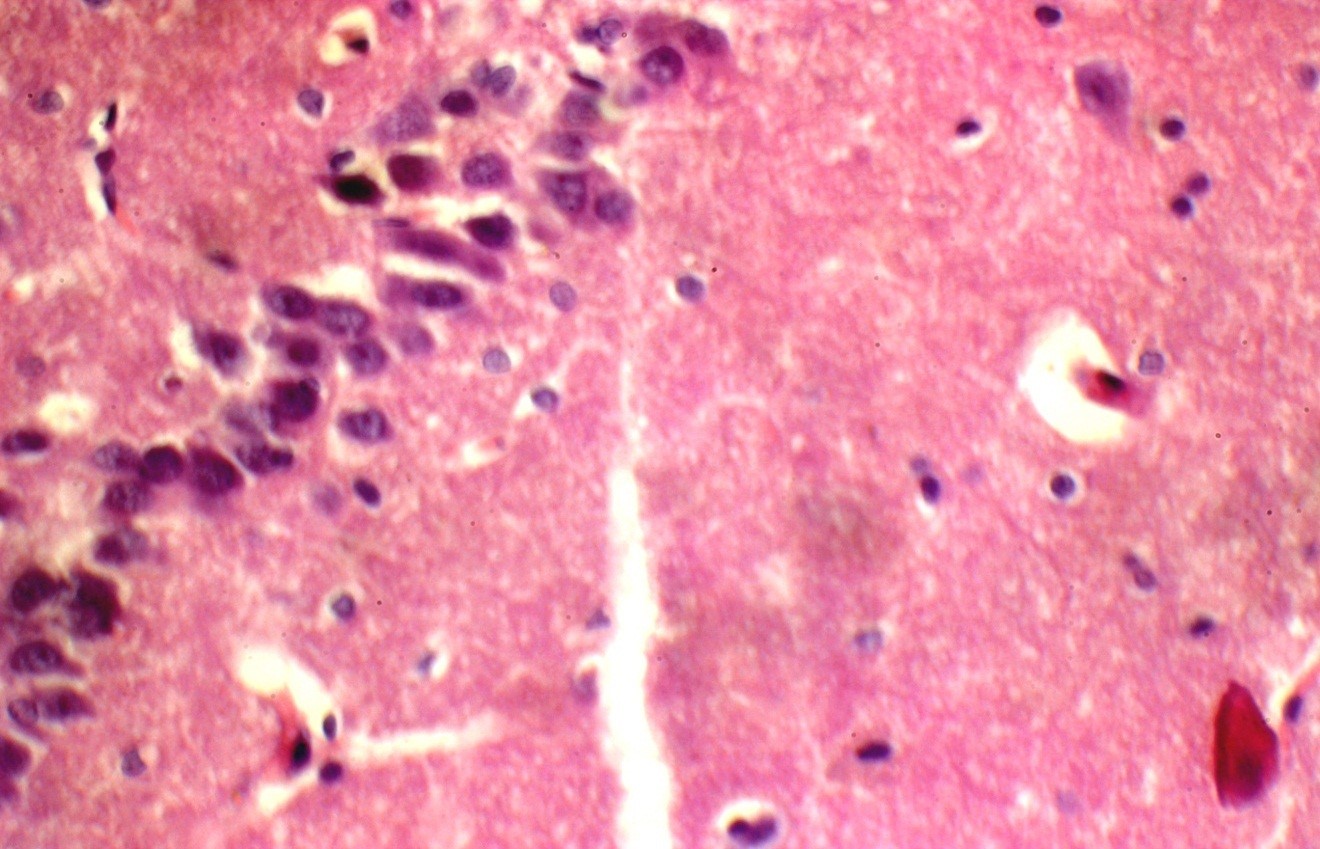

Supplement: Supplementary file 1 — (ZIP 8.38 MB) [file 210_2025_4876_MOESM1_ESM.zip › C.... Hippo.jpg]

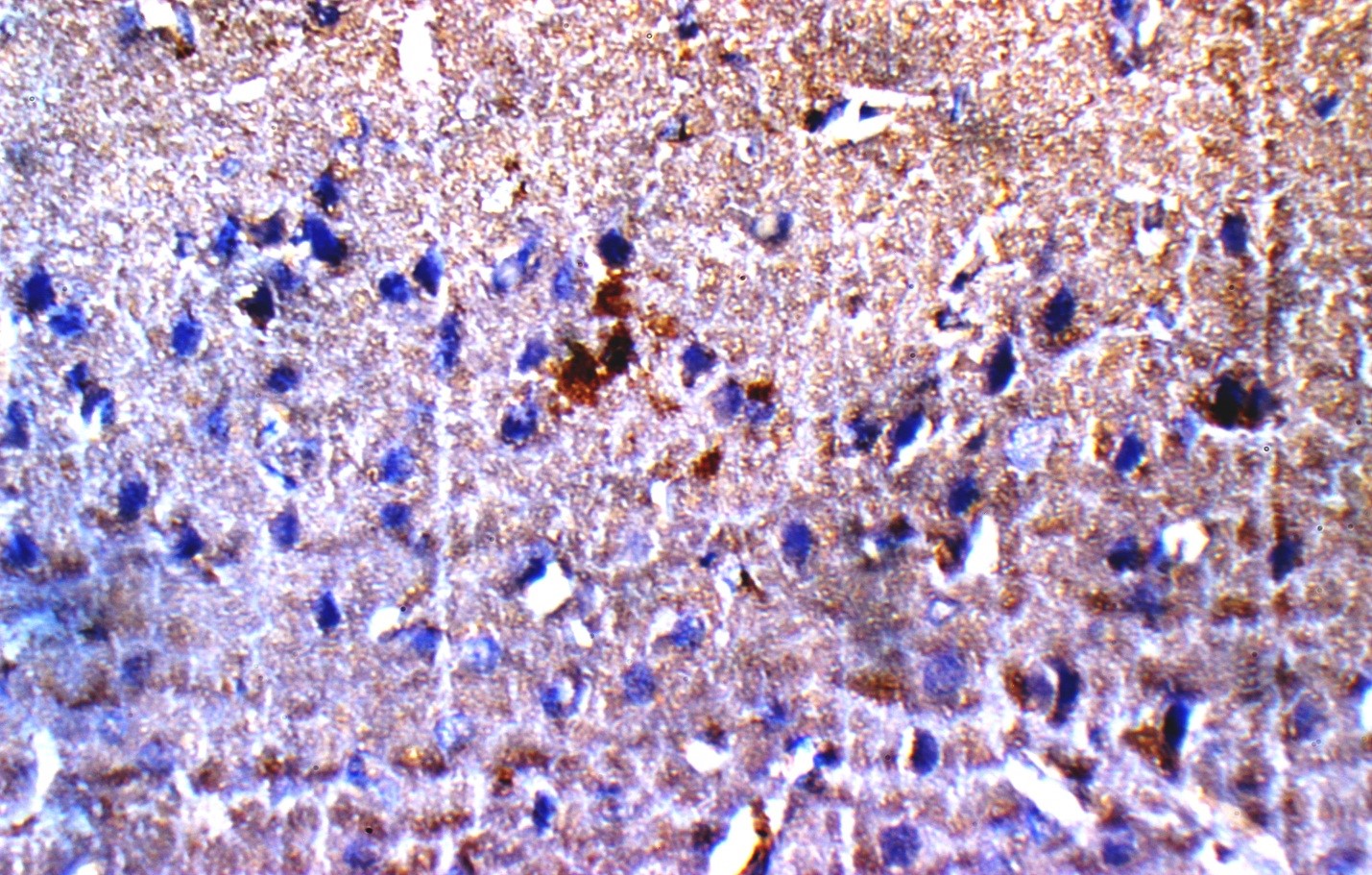

Supplement: Supplementary file 1 — (ZIP 8.38 MB) [file 210_2025_4876_MOESM1_ESM.zip › C.jpg]
